# Neurodevelopment in the First 2 Years of Life Following Prenatal Exposure to Maternal SARS-CoV-2 Infection

**DOI:** 10.1001/jamanetworkopen.2024.43697

**Published:** 2024-11-07

**Authors:** Daphne M. Vrantsidis, Marcel van de Wouw, Emily R. M. Hall, Verena Kuret, Charlie Rioux, Melanie L. Conrad, Christine Mesa, Angela Harris, Catherine Lebel, Lianne Tomfohr-Madsen, Gerald F. Giesbrecht

**Affiliations:** 1 Department of Pediatrics, University of Calgary, Calgary, Alberta, Canada; 2 Alberta Children’s Hospital Research Institute (ACHRI), Calgary, Alberta, Canada; 3 Department of Obstetrics & Gynecology, University of Calgary, Calgary, Alberta, Canada; 4 Department of Interdisciplinary Human Sciences, Texas Tech University, Lubbock; 5 Institute of Medical Psychology, Charité–Universitätsmedizin Berlin, Corporate Member of Freie Universität Berlin, Humboldt-Universität zu Berlin, Berlin, Germany; 6 Institute of Microbiology, Infectious Diseases and Immunology, Charité–Universitätsmedizin Berlin, Corporate Member of Freie Universität Berlin, Humboldt-Universität zu Berlin, Berlin, Germany; 7 National Microbiology Laboratory at the J. C. Wilt Infectious Diseases Research Centre, Public Health Agency of Canada, Winnipeg, Manitoba, Canada; 8 Department of Radiology, University of Calgary, Calgary, Alberta, Canada; 9 Department of Educational and Counselling Psychology, and Special Education, University of British Columbia, Vancouver, British Columbia, Canada; 10 Department of Psychology, University of Calgary, Calgary, Alberta, Canada; 11 Department of Community Health Sciences, University of Calgary, Calgary, Alberta, Canada

## Abstract

**Importance:**

The effects of prenatal exposure to SARS-CoV-2 infection on child development throughout the first 2 years of life are unknown.

**Objective:**

To evaluate whether prenatal exposure to SARS-CoV-2 infection was associated with child neurodevelopmental outcomes during the first 2 years of life.

**Design, Setting, and Participants:**

This cohort study used data from the longitudinal, population-based pan-Canadian Pregnancy During the COVID-19 Pandemic cohort, which recruited participants from April 2020 to July 2022. Children were categorized as exposed to prenatal SARS-CoV-2 infection if their birthing parent had a positive polymerase chain reaction test performed by a health authority or as a healthy negative comparison if their birthing parent did not have SARS-CoV-2 antibodies in their postpartum dried blood spot sample.

**Exposure:**

Prenatal SARS-CoV-2 infection.

**Main Outcomes and Measures:**

The birthing parent reported on their child’s temperament at ages 6 and 24 months, developmental milestones at ages 12 and 24 months, and social-emotional milestones at ages 12 and 24 months.

**Results:**

A total of 896 children were included, with 96 children who had been exposed to a prenatal SARS-CoV-2 infection (mean [SD] gestational age at birth, 39.20 [1.50] weeks; 45 [47%] male) and 800 were healthy negative comparisons (mean [SD] gestational age at birth, 39.47 [1.54] weeks; 388 [49%] male). In analyses of covariance adjusted for prepregnancy medical conditions and household socioeconomic status, prenatal exposure to SARS CoV-2 infection was associated with slightly higher regulatory control scores, indicating more regulation, at age 6 months (difference in means, 0.19 [95% CI, 0.02-0.36]; *P* = .03; η_p_^2^ = 0.01). No significant differences were observed for the other neurodevelopmental outcomes. In mixed models adjusted for the same covariates that aimed to examine change in outcomes over time, prenatal SARS-CoV-2 infection exposure was not associated with developmental change in any neurodevelopmental outcomes between ages 6 and 24 months.

**Conclusions and Relevance:**

In this longitudinal cohort study of multiple aspects of child neurodevelopment between ages 6 and 24 months, negligible associations between prenatal exposure to SARS-CoV-2 infection and child outcomes were observed. Follow-up research is warranted to determine whether these predominantly null effects persist into later childhood.

## Key Points

**Question:**

Is prenatal exposure to SARS-CoV-2 infection associated with child neurodevelopment throughout the first 2 years of life?

**Findings:**

In this cohort study of 896 children, repeated measures of neurodevelopmental outcomes were compared between children prenatally exposed to SARS-CoV-2 infection (confirmed by polymerase chain reaction) vs those who were not exposed (confirmed by COVID-19 antibody testing). Prenatal exposure to SARS-CoV-2 infection was associated with modestly more parent-reported infant regulatory behavior at 6 months of age but no other neurodevelopmental outcomes.

**Meaning:**

In this study, prenatal exposure to SARS-CoV-2 infection had a negligible association with child neurodevelopment during the first 2 years of life.

## Teaser

This cohort study examines whether there are differences in neurodevelopmental outcomes during the first 2 years of life among children who were exposed and unexposed to SARS-CoV-2 prenatally.

## Introduction

Exposure to viral infections during pregnancy, such as seasonal influenza and severe acute respiratory syndrome, is associated with adverse neurodevelopmental outcomes in children, including lower IQ scores, lower educational achievement, and increased risk for neurodevelopmental and neuropsychiatric disorders.^1,2,3,4^ During the COVID-19 pandemic, pregnant individuals were one of the groups most susceptible to severe medical complications due to SARS-CoV-2 infection.^5^ Although vertical transmission of SARS-CoV-2 during gestation is rare,^6,7^ the accompanying systemic inflammatory response in pregnant individuals may interfere with fundamental neurodevelopmental processes that can increase children’s risk for neurodevelopmental difficulties.^8,9,10^ Given the magnitude of the pandemic, researchers and clinicians have emphasized the need for repeated follow-up of children prenatally exposed to SARS-CoV-2 infection to better understand the impact of prenatal exposure on their neurodevelopment and long-term health.^11,12^

Most studies of the impact of prenatal SARS-CoV-2 infection exposure have only examined neurodevelopmental outcomes at a single time point during the first 18 months of life, and they have found a limited impact of prenatal exposure.^13,14,15^ A recent meta-analysis of findings from infants (aged 3 to 12 months) using the Ages and Stages Questionnaire, third edition (ASQ-3), a widely used parent-report, norm-referenced screener for neurodevelopmental milestones, found that prenatal SARS-CoV-2 infection exposure was associated with increased odds of fine motor impairment but not gross motor, communication, problem-solving, or personal-social impairments.^16^ Similarly, a second meta-analysis of children aged 0 to 17 months found prenatal SARS-CoV-2 infection exposure was associated with poorer performance on the fine motor and problem-solving domains of the ASQ-3.^13^ No significant differences were found for gross motor, communication, or personal-social scores.^17^ Critically, neurodevelopment also includes neurosensory and behavioral outcomes. In particular, child temperament, which includes reactivity to sensory stimuli and the ability to self-regulate behavior,^18^ is an important predictor of neurodevelopmental outcomes and disorders in childhood.^19,20,21,22^ Prenatal exposure to SARS-CoV-2 infection may have effects on domains of neurodevelopment unmeasured by the ASQ-3. In addition, the effects of prenatal infection exposure on infant outcomes may emerge over time.^23^ Longitudinal research into the second year of life and further into childhood is needed to address this essential gap in the literature.

Previous research has also either not included a comparison group or included a prepandemic cohort as a comparison group.^13,16^ Although children born prior to the COVID-19 pandemic do not have prenatal SARS-CoV-2 infection exposure, they may differ in important ways from children born during the pandemic. Notably, studies with a prepandemic comparison group or no comparison group have reported significant effects of prenatal SARS-CoV-2 infection exposure on neurodevelopmental outcomes, whereas studies that have included a negative (no prenatal exposure) comparison group have not found significant differences.^13,16,24^ This pattern of results emphasizes the importance of confirming the absence of SARS-CoV-2 antibodies in the negative comparison group, as misclassified controls may reduce effect sizes, leading to type II errors. More definitive evidence for the neurodevelopmental effects of prenatal exposure to SARS-CoV-2 infection can be achieved with a contemporaneous comparison group in which nonexposure is verified.

Accordingly, this study aimed to compare the neurodevelopmental outcomes of children with prenatal SARS-CoV-2 infection exposure with children without this exposure. Neurodevelopmental outcome domains included temperament at ages 6 months (Infant Behavior Questionnaire–Revised, Very Short Form [IBQ-R-VSF]) and 24 months (Early Childhood Behavior Questionnaire [ECBQ]), developmental milestones at ages 12 and 24 months (ASQ-3), and socioemotional milestones at ages 12 and 24 months (Ages and Stages: Social-Emotional, second edition [ASQ:SE-2]). A key strength of this study was the repeated measurement of child outcomes across the first 2 years of life, allowing us to examine both mean scores at multiple time points and developmental change over time in these outcomes. Repeated measurements provide an early indication of whether children with a prenatal SARS-CoV-2 infection exposure have slower neurodevelopment over time relative to children without exposure. This is important for determining whether neurodevelopmental effects of prenatal exposure are more likely to emerge at older ages or over time.

## Methods

### Study Design and Participants

Participants were from the pan-Canadian longitudinal Pregnancy During the COVID-19 Pandemic Study,^25,26^ a prospectively recruited pregnancy cohort designed to study the impact of the COVID-19 pandemic on pregnant individuals and their children throughout Canada (Figure). Pregnant individuals were eligible if they lived in Canada, were 17 years or older, were able to read and write in English or French, and were no more than 35 weeks’ gestation at enrollment. No incentive was offered for enrollment. Participants were entered into monthly draws to win one $100 gift card for each follow-up survey they completed between enrollment and when their infant was aged 9 months. Participants also received a $25 gift card for completing the follow-up surveys when the child was aged 1 and 2 years. The University of Calgary Conjoint Health Research Ethics Board approved this study. Participants provided informed consent at enrollment. This report follows the Strengthening the Reporting of Observational Studies in Epidemiology (STROBE) guidelines.^27^

**Figure.**
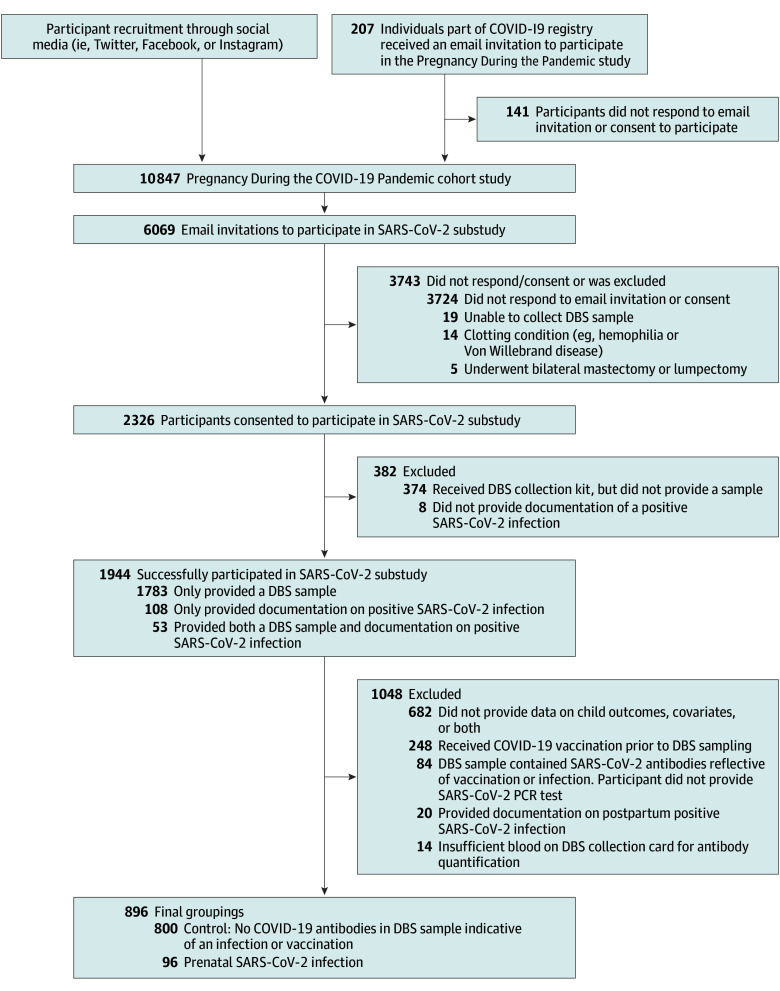
Flowchart Outlining Participant Inclusion and Exclusion Groupings DBS indicates dried blood sample; PCR, polymerase chain reaction.

Individuals across Canada were recruited between April 2020 and July 2022 using social media advertisements. Administrative health data and the Canadian Surveillance of COVID-19 in Pregnancy: Epidemiology, Maternal and Infant Outcomes surveillance system were also used to identify individuals infected with SARS-CoV-2 during pregnancy who were invited to participate in the study. Data on infection status and antibody levels were prospectively collected on pregnant individuals with a SARS-CoV-2 infection between April 2020 and December 2022.

The SARS-CoV-2 exposure group was defined as children born to pregnant individuals who had a positive SARS-CoV-2 polymerase chain reaction (PCR) test during pregnancy performed by a health authority. Documentation of a positive test was provided to the study team, whereupon the team confirmed the inclusion criteria. Pregnant individuals who provided documentation of a positive test self-reported details about their infection severity and symptoms via an online questionnaire. The SARS-CoV-2 negative comparison group was defined as children of pregnant individuals who self-reported that they had not experienced a flu-like illness during their pregnancy, who did not have a positive PCR test during pregnancy, who had not received a SARS-CoV-2 vaccination before or during pregnancy, and whose dried blood spot (DBS) sample did not contain any SARS-CoV-2 antibodies that would indicate either a previous vaccination or SARS-CoV-2 infection. DBS samples were collected between 1 and 12 months post partum. Pregnant individuals were excluded from DBS collection if they had contraindications including a clotting condition, such as hemophilia or Von Willebrand disease, or if they had undergone a bilateral mastectomy or lumpectomy. Since all births in the exposure group were singletons, participants who had births with multiple children were excluded from the negative comparison group.

Questionnaires administered at study enrollment measured self-reported demographic (eg, race and ethnicity) and socioeconomic status (SES) characteristics. Racial and ethnic groups included Black, East Asian, Hispanic or Latina, Indigenous, Southeast Asian, South Asian, West Asian, and White. A category for participants who selected multiple racial and ethnic groups or who did not self-identify with any of the aforementioned groups was also created.

Birth outcomes were self-reported at the first postpartum follow-up survey (1 to 6 months’ post partum). Participants reported on their child’s temperament at 6 and 24 months of age and on their developmental and socioemotional milestones at 12 and 24 months of age. Research Electronic Data Capture (REDCap) was used to obtain participant consent and administer questionnaires.^28,29^

### DBS Sample Collection and SARS-CoV-2 Antibody Analyses

SARS-CoV-2 antibodies were measured from DBS samples that participants self-collected using methods developed by the COVID-19 Immunity Task Force^30^ and Statistics Canada Canadian COVID-19 Antibody and Health Survey.^31^ DBS collection materials were mailed to participants. Participants collected the DBS sample at home and mailed the sample back to the study team. For DBS collection, a lancet was used to pierce a finger, which was then blotted onto filter paper. The sample was dried for 3 hours and packaged by the participant using a gas-impermeable bag containing desiccant, then shipped at room temperature to our laboratory. DBS antibody testing was performed at the National Microbiology Laboratory using the multiplex BioPlex 2200 SARS-CoV-2 immunoglobulin G (IgG) assay capable of differentiating IgG antibodies against the receptor-binding domain (RBD), spike 1 (S1), and nucleocapsid (N). In samples collected at 1 to 28 days after symptom onset, this assay achieved the following overall sensitivities: RBD, 96.4% (95% CI, 91.9%-98.5%); SI, 97.9% (95% CI, 93.9%-99.4%); and N, 92.1% (95% CI, 86.5%-95.6%).^32^ Similarly, overall specificity for RBD was 98.9% (95% CI, 94.7%-100%); SI, 100% (95% CI, 96.4%-100%); and N, 100% (95% CI, 96.4%-100%). Studies have demonstrated detectable antibodies 8 months after SARS-CoV-2 infection.^33^

### Child Neurodevelopmental Outcomes

Temperament was measured when children were aged 6 months using the IBQ-R-VSF and 24 months using the ECBQ. The IBQ-R-VSF is a 37-item parent-report measure of 3 broad dimensions of temperament in infants ages 3 to 12 months: positive affectivity/surgency, regulatory capacity/orienting, and negative affectivity.^34,35^ The IBQ-R-VSF has strong psychometric properties and is widely used in the child development literature.^36,37^ The ECBQ is comparable with the IBQ-R-VSF, but designed for older children (aged 18 to 36 months).^35^ To limit participant burden, only the 12-item negative affectivity ECBQ subscale was administered. For both questionnaires, participants reported their observations of specific child temperament behaviors in the past week using a 7-point Likert scale ranging from never to always. Scores on each item were summed to create scores for each dimension. Dimensional scores ranged from 1 to 7, with higher scores reflecting stronger evidence of each dimension.

Developmental milestones were measured when children were aged 12 and 24 months using the ASQ-3, a 30-item measure of child development in 5 domains: communication, gross motor, fine motor, problem-solving, and personal-social skills.^38^ The American Academy of Pediatrics identifies the ASQ-3 as a high-quality tool for use in clinical practice to screen for delayed developmental milestones in children.^39^ Participants rated each item as yes (10 points), sometimes (5 points), or not yet (0 points) based on their child’s usual behavior. A total score for each domain was obtained by summing the 6 items assessing that domain. Lower scores indicate more developmental problems.

Socioemotional milestones were measured when children were aged 12 and 24 months using the ASQ:SE-2, a 27-item measure (31 items at 24 months) of 7 areas of socioemotional development: self-regulation, compliance, social-communication, adaptive functioning, autonomy, affect, and interaction with people.^40^ Participants rated each item on a scale ranging from often or always (0 points), sometimes (5 points), or rarely or never (10 points) based on their child’s usual behavior. A total score indexing overall socioemotional problems was calculated by summing all 27 items. Higher scores indicate more socioemotional problems.

### Statistical Analysis

Statistical analyses were conducted using SPSS statistical software version 29.0.0.0 (IBM Corp). Separate analyses were conducted for each outcome. For all analyses, a 2-tailed *P* < .05 indicated statistical significance. Power analyses were conducted for the primary outcome examining neurodevelopmental differences for infants exposed and not exposed to SARS-CoV-2 infection prenatally. We had 80% power to detect a less than 0.16-SD difference in each neurodevelopmental outcome, equivalent to an η_p_^2^ = 0.15, using a 2-sided test and an α = .05.

Sociodemographic comparisons between the prenatal exposure and negative comparison groups were conducted using χ^2^ for categorical outcomes and *t* tests for continuous outcomes. To test for group differences in child outcomes, we conducted analyses of covariance (ANCOVAs) with robust standard errors and adjusted for covariates. We created a causal directed acyclic graph to identify potential confounders (eFigure in Supplement 1).^41,42^ Prepregnancy medical conditions that increased the risk for SARS-CoV-2 infection and household SES were adjusted for in all analyses. These confounders were identified a priori based on (1) associations with SARS-CoV-2 infection during pregnancy and child neurodevelopmental outcomes and (2) temporally preceding both the exposure and outcomes.^43,44^ Prepregnancy medical conditions (ie, diabetes, heart disease, hypertension, celiac disease, hypothyroidism, hyperthyroidism, ulcerative colitis, Crohn disease, asthma, and obesity; coded as 0 for no medical conditions and 1 for 1 or more medical conditions) and household SES (a mean consisting of the *z* scores of pregnant individual’s education, household income, and food insecurity) were self-reported at enrollment.

Mixed models were used to examine whether prenatal SARS-CoV-2 infection exposure was associated with developmental change in negative affectivity scores from ages 6 to 24 months and ASQ-3 and ASQ:SE-2 scores from ages 12 to 24 months. Prenatal exposure status, time of outcome measurement, the interaction between exposure status and measurement time point, and covariates were included as fixed effects. The time point of outcome measurement was centered at 6 months for negative affectivity and 12 months for ASQ measures. To account for repeated measurement of the outcome, participants were included as a random effect. Random intercepts were used to allow for variability in initial scores. Robust standard errors and an identity matrix were used for random effects. Maximum likelihood estimation was used to permit statistical tests of both fixed and random effects.^45^

We ran 3 supplemental analyses. First, we ran exploratory analyses examining whether child sex moderated the association between SARS-CoV-2 exposure and child outcomes by adding the main effect of child sex and the child sex × infection status interaction term to the ANCOVAs and mixed models. Second, we ran ANCOVAs with robust standard errors to examine whether (1) trimester of exposure and (2) infection severity were associated with child outcomes for the prenatal exposure group. Following World Health Organization guidelines, infection severity was categorized as 1, asymptomatic; 2, mild symptoms not requiring medical attention; 3, symptomatic, assistance needed; 4, hospitalized but no oxygen therapy; and 5, hospitalized and received oxygen therapy.^46^ Third, we examined the sensitivity of our findings to missing data by rerunning all analyses using multiple imputation (50 imputations) to account for selective dropout.^46^

## Results

### Sample Characteristics

Ninety-six children (mean [SD] gestational age at birth, 39.20 [1.50] weeks; 45 [47%] male) had prenatal SARS-CoV-2 infection exposure confirmed by a PCR test. The 800 healthy negative comparison children (mean [SD] gestational age at birth, 39.47 [1.54] weeks; 388 [49%] male) had a birthing parent with no SARS-CoV-2 antibodies in their DBS samples. Birthing parents who had a SARS-CoV-2 infection had lower education than birthing parents in the comparison group. Groups did not differ on any other parent or child sociodemographic or clinical characteristic, including gestational age, birth weight, or child age at assessment. Mean (SD) gestational age at the time of SARS-CoV-2 infection was 20.70 (9.30) weeks. The eAppendix in Supplement 1 reports additional infection details. Most participants were symptomatic (95 [99%]) and experienced lingering symptoms (52 [55%]). Only a small portion were hospitalized (5 [5%]). Birthing parent and child sociodemographic and clinical characteristics are reported in Table 1. Descriptive statistics for the outcome measures are reported in Table 2. eTable 1 in Supplement 1 reports descriptive statistics for clinical cutoffs on the ASQ.

**Table 1.** Sample Characteristics

Characteristic	Participants, No. (%)		χ^2^	*P* value
	Prenatal SARS-CoV-2 exposure (n = 96)	No exposure (n = 800)		
**Mother**				
Age at delivery, mean (SD) [range], y	32.50 (4.00) [23.10 to 42.80]	33.20 (3.90) [21.83 to 49.33]	*t*, −1.48 (95% CI, −1.50 to 0.22)	.14
Race and ethnicity				
Black	2 (2)	8 (1)	8.43	.59
East Asian	4 (4)	21 (3)		
Hispanic/Latina	3 (3)	18 (2)		
Indigenous	1 (1)	22 (3)		
Southeast Asian	2 (2)	3 (0.40)		
South Asian	0	21 (3)		
West Asian	0	6 (1)		
White	80 (83)	652 (82)		
Multiple ethnicities or other^a^	2 (2)	44 (5)		
Missing	2 (2)	5 (1)		
Education				
High school diploma or less	10 (10)	34 (0.30)	18.86	.04
Trade or technical degree	22 (23)	133 (17)		
Bachelor’s degree	45 (47)	350 (44)		
Graduate degree (MSc, MD, PhD)	17 (19)	283 (35)		
Missing	2 (2)	0		
Annual household income, $				
69 999	15 (15)	134 (18)	3.59	.89
70 000-99 999	13 (14)	115 (14)		
100 000-124 999	17 (18)	168 (21)		
125 000-149 999	16 (17)	111 (14)		
150 000-174 999	17 (18)	108 (14)		
175 000-199 999	8 (8)	72 (9)		
≥200 000	8 (8)	92 (12)		
Missing	2 (2)	0		
Food insecurity				
Often or sometimes	5 (5)	27 (3)	1.10	.58
Prepregnancy medical conditions				
Any	30 (31)	230 (29)	0.26	.61
Diabetes, type 1	0	0		
Diabetes, type 2	0	1 (<1)		
Heart disease	0	7 (1)		
Hypertension	2 (2)	8 (1)		
Celiac disease	0	13 (2)		
Hypothyroidism	4 (4)	50 (6)		
Hyperthyroidism	0	7 (1)		
Ulcerative colitis	1 (1)	3 (<1)		
Crohn disease	1 (1)	2 (<1)		
Asthma	13 (14)	87 (11)		
Obesity	20 (21)	159 (20)		
**Child**				
Gestational age at birth, mean (SD) [range], wk	39.20 (1.50) [33.10 to 41.60]	39.47 (1.54) [26.57 to 42.43]	*t*, −1.08 (95% CI, −0.49 to 0.14)	.28
Preterm birth^b^	2 (2)	43 (5)	1.84	.18
Missing	3 (3)	5 (1)	NA	NA
Birth weight, mean (SD) [range], g	3360 (435) [2282 to 4617]	3445 (517) [750 to 5160]	*t*, −1.77 (95% CI, −180 to 10)	.08
Child sex				
Male	45 (47)	388 (49)	0.01	.93
Female	51 (53)	412 (51)		
Missing	4 (4)	0	NA	NA
Vaginal delivery	66 (69)	541 (68)	0.45	.50
Missing	7 (7)	33 (4)	NA	NA
Age at 6-mo assessment, mean (SD) [range], mo	6.19 (0.37) [5.91 to 7.75]	6.11 (0.26) [5.91 to 8.11]	*t*, 1.69 (95% CI, −0.01 to 0.17)	.10
Age at 12-mo assessment, mean (SD) [range], mo	12.23 (0.59) [11.56 to 15.67]	12.18 (0.48) [11.37 to 15.97]	*t*, 0.89 (95% CI, −0.06 to 0.15)	.37
Age at 24-mo assessment, mean (SD) [range], mo	23.54 (0.84) [23.00 to 28.00]	23.47 (0.70) [23.00 to 28.00]	*t*, 0.69 (95% CI, −0.11 to 0.24)	.49

Abbreviation: NA, not applicable.

^a^ Other includes individuals who did not self-identify with any of the racial or ethnic categories listed.

^b^ Preterm birth was defined as gestational age younger than 37 weeks.

**Table 2.** Neurodevelopmental Outcomes for Children Exposed Prenatally to SARS-CoV-2 and a Negative Exposure Comparison Group^a^

Outcome (age)	Prenatal SARS-CoV-2 exposure		No exposure		B (95% CI)	*P* value	η_p_^2^
	No.	Mean (SD)	No.	Mean (SD)			
**Temperament**							
IBQ-R-VSF surgency (6 mo)	60	4.76 (0.77)	640	4.67 (0.81)	0.09 (−0.13 to 0.30)	.43	0.00
IBQ-R-VSF regulation (6 mo)	60	5.62 (0.53)	640	5.42 (0.65)	0.19 (0.02 to 0.36)	.03	0.01
IBQ-R-VSF negative affectivity (6 mo)	60	3.58 (1.07)	633	3.59 (0.97)	−0.01 (−0.27 to 0.25)	.93	0.00
ECBQ negative affectivity (24 mo)	68	2.55 (0.59)	687	2.58 (0.64)	−0.05 (−0.21 to 0.11)	.56	0.00
**Developmental milestones**							
ASQ-3 communication (12 mo)	80	48.48 (9.98)	716	47.70 (12.07)	0.87 (−1.88 to 3.61)	.54	0.00
ASQ-3 gross motor (12 mo)	79	45.95 (15.91)	716	45.40 (16.04)	0.59 (−3.14 to 4.33)	.77	0.00
ASQ-3 fine motor (12 mo)	80	51.88 (7.73)	713	52.71 (7.97)	−0.79 (−2.63 to 1.05)	.40	0.00
ASQ-3 problem-solving (12 mo)	79	47.41 (10.53)	711	47.92 (10.90)	−0.44 (−2.97 to 2.09)	.73	0.00
ASQ-3 personal-social (12 mo)	80	43.06 (13.51)	712	44.28 (12.20)	−1.21 (−4.07 to 1.65)	.41	0.00
ASQ-3 communication (24 mo)	65	49.92 (11.47)	685	50.15 (13.00)	0.24 (−3.02 to 3.49)	.89	0.00
ASQ-3 gross motor (24 mo)	79	53.15 (7.43)	686	52.63 (9.29)	0.61 (−1.73 to 2.94)	.61	0.00
ASQ-3 fine motor (24 mo)	65	49.92 (7.04)	685	51.06 (8.16)	−0.93 (−2.98 to 1.13)	.38	0.00
ASQ-3 problem-solving (24 mo)	65	45.15 (8.75)	683	46.88 (9.69)	−1.68 (−4.14 to 0.77)	.18	0.00
ASQ-3 personal-social (24 mo)	65	48.46 (9.43)	685	49.83 (8.91)	−1.08 (−3.36 to 1.19)	.35	0.00
**Socioemotional milestones**							
ASQ:SE-2 (12 mo)	65	25.19 (17.14)	707	27.51 (18.95)	−2.49 (−6.84 to 1.85)	.26	0.00
ASQ:SE-2 (24 mo)	65	25.69 (15.58)	675	25.56 (20.14)	−0.58 (−5.55 to 4.38)	.82	0.00

Abbreviations: ASQ:SE-2, Ages and Stages Questionnaire: Social-Emotional, second edition; ASQ-3; Ages and Stages Questionnaire, third edition; ECBQ, Early Childhood Behavioral Questionnaire; IBQ-R-VSF, Infant Behavior Questionnaire–Revised, Very Short Form.

^a^ Results for full models, including covariates, are reported in eTable 2 in Supplement 1. All analyses were adjusted for prepregnancy medical conditions that increased the risk for SARS-CoV-2 infection and household socioeconomic status.

### ANCOVAs

In analyses adjusted for prepregnancy medical conditions and household SES, children exposed to SARS-CoV-2 infection had higher IBQ-R-VSF regulation scores at 6 months than comparison children (difference in means, 0.19 [95% CI, 0.02-0.36; *P* = .03; η_p_^2^ = 0.01), indicating more regulatory behavior in everyday contexts (Table 2; eTable 2 in Supplement 1). Children in the exposure group did not significantly differ from children in the comparison group on any other outcome measures.

### Mixed Models

In analyses adjusted for prepregnancy medical conditions and household SES, group membership was not significantly associated with the intercepts or slopes of any neurodevelopmental outcomes (Table 3). eTable 3 in Supplement 1 reports results with covariates.

**Table 3.** Developmental Change in Child Neurodevelopmental Outcomes^a^

Outcome	b (SE) [95% CI]	*t*	*P* value
**Temperament: negative affectivity**			
Intercept	3.62 (0.04) [3.55 to 3.70]	96.01	<.001
Exposure status	−0.03 (0.11) [−0.24 to 0.19]	−0.24	.81
Time point	−0.06 (0.00) [−0.06 to −0.05]	−26.88	<.001
Exposure × time point	−0.00 (0.01) [−0.02 to 0.01]	−0.38	.70
**Developmental milestones**			
Communication			
Intercept	47.26 (0.54) [46.20 to 48.33]	87.02	<.001
Exposure status	0.99 (1.44) [−1.83 to 3.81]	0.69	.49
Time point	0.21 (0.04) [0.12 to 0.29]	4.71	<.001
Exposure × time point	−0.05 (0.15) [−0.34 to 0.24]	−0.34	.73
Gross motor			
Intercept	45.78 (0.58) [44.64 to 46.92]	78.85	<.001
Exposure status	0.60 (1.55) [−2.43 to 3.63]	0.39	.70
Time point	0.62 (0.05) [0.53 to 0.71]	13.18	<.001
Exposure × time point	−0.05 (0.16) [−0.36 to 0.26]	−0.29	.77
Fine motor			
Intercept	52.71 (0.35) [52.03 to 53.40]	150.57	<.001
Exposure status	−0.74 (0.94) [−2.59 to 1.10]	−0.79	.43
Time point	−0.13 (0.03) [−0.19 to −0.07]	−4.24	<.001
Exposure × time point	0.01 (0.10) [−0.20 to 0.21]	0.05	.96
Problem-solving			
Intercept	47.70 (0.45) [46.81 to 48.59]	105.10	<.001
Exposure status	−0.40 (1.21) [−2.78 to 1.97]	−0.33	.74
Time point	−0.08 (0.04) [−0.16 to −0.01]	−2.16	.03
Exposure × time point	−0.11 (0.13) [−0.36 to 0.14]	−0.85	.40
Personal-social			
Intercept	44.03 (0.48) [43.09 to 44.96]	92.53	<.001
Exposure status	−1.14 (1.27) [−3.62 to 1.35]	−0.90	.37
Time point	0.47 (0.04) [0.39 to 0.55]	11.48	<.001
Exposure × time point	−0.04 (0.14) [−0.31 to 0.23]	−0.30	.77
Socioemotional milestones: social-emotional			
Intercept	29.14 (0.86) [27.45 to 30.82]	33.98	<.001
Exposure status	−2.65 (2.22) [−7.00 to 1.70]	−1.20	.23
Time point	−0.15 (0.06) [−0.26 to −0.04]	−2.65	.008
Exposure × time point	0.17 (0.19) [−0.22 to 0.55]	0.85	.39

^a^ Results for full models, including covariates, are reported in eTable 3 in Supplement 1. Prepregnancy medical conditions that increased the risk for SARS-CoV-2 infection and household socioeconomic were adjusted for in all analyses.

### Supplemental Analyses

The interaction between child sex and infection exposure status was not significant for neurodevelopmental outcomes at any time point or changes in neurodevelopment over time (eTable 4 in Supplement 1). Trimester of exposure and infection severity were not significantly associated with any neurodevelopmental outcomes at ages 6, 12, or 24 months (eTable 5 in Supplement 1). Missing data on the IBQ-R-VSF, ASQ-3 gross motor scale at 12 months, ECBQ, and ASQ-3 and ASQ:SE-2 measures at 24 months were associated with exposure status (eTable 6 in Supplement 1). Results were unchanged when analyses were rerun with imputed data (eTable 6 in Supplement 1).

## Discussion

This study examined the association between prenatal SARS-CoV-2 infection exposure and children’s neurodevelopmental outcomes (temperament, developmental milestones, and socioemotional milestones) at ages 6, 12, and 24 months and developmental change in outcomes between ages 6 and 24 months. Prenatal exposure was associated with more regulatory behavior at age 6 months (temperament) but was unrelated to all other outcomes at each time point and developmental change. Our results suggest that the association between prenatal SARS-CoV-2 infection exposure and children’s neurodevelopmental outcomes was negligible across the first 2 years of life.

In this cohort, prenatal SARS-CoV-2 infection exposure was associated with more infant regulatory behavior at age 6 months. Greater regulatory ability at age 6 months is associated with better attentional ability and is generally considered a strength. Nevertheless, the effect size was very small. Although unexpected, this result may support research theorizing that in utero exposure to SARS-CoV-2 infection leads to accelerated maturation, as has been found for other adverse prenatal exposures like stress.^47,48^ Consistent with this suggestion, on many of the ASQ-3 measures at ages 12 and 24 months, fewer children exposed to SARS-CoV-2 infection prenatally met the cutoff for clinical referral (ie, scored 2 SD below the mean) compared with unexposed children, although personal-social scores at 12 months represent a notable exception. These differences should be interpreted with caution as they are preliminary, and the sample and cell sizes are small. Nonetheless, one interpretation of them is in support of accelerated maturation. Some research has found that self-regulatory abilities in infancy are positively associated with developmental milestones like communication and motor skills.^49,50^ This suggests that more infant regulatory behavior at age 6 months may be driving developmental differences at ages 12 and 24 months. Importantly, accelerated early development of regulatory behavior may be maladaptive long term, as it is linked to the development of mood and anxiety disorders.^48^ We did not administer the full ECBQ at age 24 months, so we were unable to examine whether these differences persisted into toddlerhood. Nonetheless, we are continuing to follow up these children until age 8 years, which will allow us to examine whether (1) this early difference in regulatory behavior is meaningfully associated with long-term psychosocial and neurodevelopmental outcomes and (2) whether the comparatively lower rate of exposed children meeting criteria for clinical referrals on the ASQ-3 persists across childhood.

Prenatal SARS-CoV-2 exposure was not association with any other neurodevelopmental outcome at individual time points or developmental change between ages 6 and 24 months. These null findings strengthen a growing body of literature comparing children with prenatal SARS-CoV-2 infection exposure with negative controls between birth and 17 months of age, which found minimal impacts of prenatal exposure on child outcomes, primarily assessed using the ASQ-3.^13,16^ Our results extend these null findings to an older age group (24 months) and contradict the suggestion that the adverse effects of prenatal SARS-CoV-2 infection exposure on neurodevelopment may emerge in toddlerhood.^23^ Additional studies using in-person assessments are needed to confirm these findings.

In addition, the present results conflict with results of studies examining differences in ASQ-3 scores between pandemic and prepandemic cohorts.^13,16^ Unlike other viral infections (eg, Zika virus),^51,52^ SARS-CoV-2 infection has a low rate of vertical transmission, so it is unlikely to directly affect fetal development.^53,54^ Therefore, previously reported adverse impacts of the COVID-19 pandemic on child neurodevelopmental outcomes may be driven by cohort effects and psychosocial changes that occurred as a result of the pandemic, including increased parental stress during and after pregnancy,^16^ social isolation,^55^ and reduced parental engagement and responsiveness,^56,57^ rather than exposure to SARS-CoV-2 infection in utero. Caregiver and teacher investment in activities that promote neurocognitive and social development (eg, shared reading) are likely to be beneficial in offsetting the adverse impact of the COVID-19 pandemic on neurodevelopment.^58^

### Limitations

This study has limitations. Parent-report measures of child neurodevelopment reflect parents’ perceptions of child development. More research with objective neurodevelopment measures will address this limitation.^59,60,61^ Not all participants who were invited to provide a PCR confirmation, DBS sample, or child development data provided these data, which may have resulted in selection bias and reduced the generalizability of the results. Consistent with the sociodemographic characteristics of Canada, the sample was predominantly White and relatively well educated and few participants had food insecurity, limiting the generalizability of the present findings.^62,63^ Additional research is needed addressing how prenatal SARS-CoV-2 infection exposure interacts with social determinants of health (eg, access to resources, belonging to a historically marginalized group) to impact neurodevelopmental outcomes.^64^

## Conclusions

This study found that prenatal SARS-CoV-2 exposure had a negligible association with child neurodevelopment between 6 and 24 months of age. Follow-up research is warranted to evaluate whether these predominantly null effects persist into later childhood.

## Notes

Supplement 1. **eFigure.** Directed Acyclic Graph **eAppendix.** Additional SARS-CoV-2 Infection **eTable 1.** Number of Children Reaching Clinical Cutoffs on Each ASQ-3 and ASQ-SE:2 Measure at Ages 12 and 24 Months **eTable 2.** Full Model Results, Including Covariates, for Neurodevelopmental Outcomes in Children Exposed Prenatally to SARS-CoV-2 Infection and Those Not Exposed Prenatally **eTable 3.** Results, Including Covariates, for Developmental Change in Children’s Neurodevelopmental Outcomes **eTable 4.** Results Examining Interactions Between Child Sex and Exposure Status **eTable 5.** Analyses by Trimester and Severity of Exposure **eTable 6.** Results Based on Imputed Data

Supplement 2.
Data Sharing Statement
