# Supplementary material for: Neurodevelopment in the First 2 Years of Life Following Prenatal Exposure to Maternal SARS-CoV-2 Infection
Source: JAMA Netw Open. 2024 Nov 7;7(11):e2443697. doi: 10.1001/jamanetworkopen.2024.43697 (PMC11544495; doi:10.1001/jamanetworkopen.2024.43697)
Supplement: Supplement 1. — eFigure. Directed Acyclic Graph eAppendix. Additional SARS-CoV-2 Infection eTable 1. Number of Children Reaching Clinical Cutoffs on Each ASQ-3 and ASQ-SE:2 Measure at Ages 12 and 24 Months eTable 2. Full Model Results, Including Covariates, for Neurodevelopmental Outcomes in Children Exposed Prenatally to SARS-CoV-2 Infection and Those Not Exposed Prenatally eTable 3. Results, Including Covariates, for Developmental Change in Children’s Neurodevelopmental Outcomes eTable 4. Results Examining Interactions Between Child Sex and Exposure Status eTable 5. Analyses by Trimester and Severity of Exposure eTable 6. Results Based on Imputed Data [file jamanetwopen-e2443697-s001.pdf]

Supplemental Online Content

Vrantsidis DM, van de Wouw M, Hall EM, et al. Neurodevelopment in the first 2 years of life following prenatal exposure to maternal SARS-CoV-2 infection. *JAMA Netw Open.* 2024;7(11):e2443697. doi:10.1001/jamanetworkopen.2024.43697

eFigure. Directed Acyclic Graph

eAppendix. Additional SARS-CoV-2 Infection

eTable 1. Number of Children Reaching Clinical Cutoffs on Each ASQ-3 and ASQ:SE-2 Measure at Ages 12 and 24 Months

eTable 2. Full Model Results, Including Covariates, for Neurodevelopmental Outcomes in Children Exposed Prenatally to SARS-CoV-2 Infection and Those Not Exposed Prenatally

eTable 3. Results, Including Covariates, for Developmental Change in Children's Neurodevelopmental Outcomes

eTable 4. Results Examining Interactions Between Child Sex and Exposure Status

eTable 5. Analyses by Trimester and Severity of Exposure

eTable 6. Results Based on Imputed Data

This supplemental material has been provided by the authors to give readers additional information about their work.

© 2024 Vrantsidis DM et al. *JAMA Network Open.*

eFigure. Directed Acyclic Graph

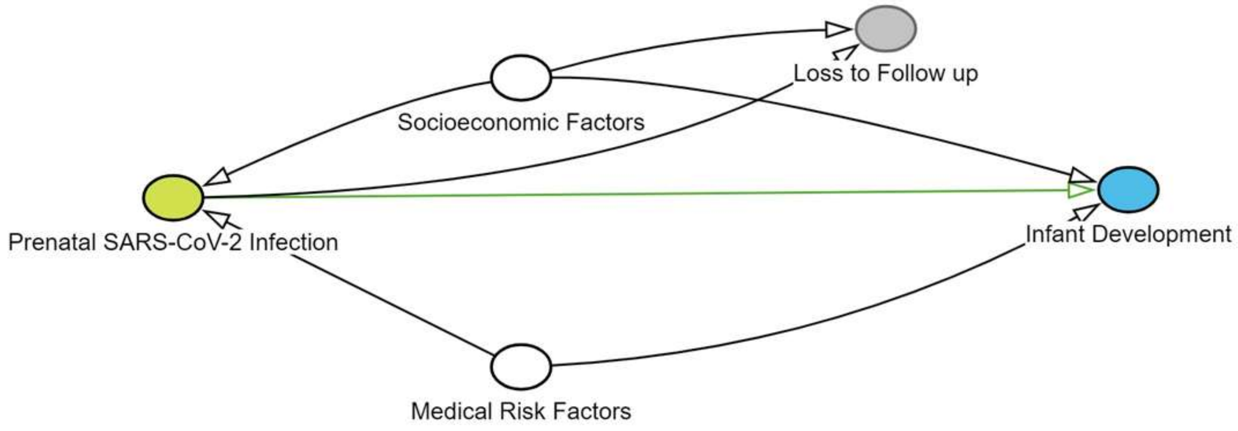

A causal directed acyclic graph (DAG) was constructed using daggity.net to identify potential confounders. Socioeconomic factors (a standardized composite of education, household income, and food insecurity) and medical risk factors that are known to associate with SARS-CoV-2 infection were identified as confounders.

© 2024 Vrantsidis DM et al. *JAMA Network Open.*

eAppendix. Additional SARS-CoV-2 Infection

Stability of SARS-CoV-2 infection antibodies

To determine whether the time delay between infection and DBS collection was associated with antibody levels, we conducted Spearman correlations for the number of weeks between the self-reported infection and DBS collection. There was a significant negative correlation between number of weeks and nucleocapsid IgG levels (r_s=-0.384, *p*=0.03), whereas the correlations for RBD and S1 IgG levels were not significant.

Prenatal COVID-19 Symptoms Severity Information

Antibody data is from all SARS-CoV-2 infected participants who provided a DBS sample (n=53).

	M (SD, range)
Gestational age at infection (weeks)	20.70 (9.50, 1 – 40)
Self-reported symptom severity compared to a normal flu (score 1-100)	46.40 (26.20, 1 – 100)
How many days did the symptoms last?	11.80 (10.50, 2 – 56)
How many symptoms did the participant experience?	5.90 (3.20, 0 – 14)
If the participant experienced lingering symptoms, how many weeks did the longest lingering symptoms last?	9.90 (16.20, 2 – 99)
If the participant experienced lingering symptoms, how many lingering symptoms did you experience?	1.10 (1.70, 0 – 8)
If the participant experienced lingering symptoms, how much have these lingering symptoms due to COVID-19 infection during pregnancy interfered with your daily life? (Score 1-100)	24.30 (30.10, 0 – 95)
If the participant was hospitalized, how many days were they in the hospital?	3.20 (2.00, 1 – 5)
RBD IgG (binding antibody units / mL)	2657 (4875, 27 – 21674)
S1 IgG (binding antibody units / mL)	2988 (4639, 56 – 18191)
Nucleocapsid IgG (binding antibody units / mL)	107 (202, 11 – 1156)
Time between infection and DBS sampling date (weeks)	22.10 (15.90, 2.10 – 70.30)
	n (%)
Trimester of infection:	
First	21 (22%)
Second	45 (47%)
Third	30 (31%)
Symptoms:	
None, infection was asymptomatic	1 (1%)
Fever	32 (33%)
Chills	42 (44%)
Headache	70 (73%)

© 2024 Vrantsidis DM et al. *JAMA Network Open.*

Cough	61 (64%)
Difficulty breathing	18 (19%)
Shortness of breath at rest	24 (25%)
Chest pain	8 (8%)
Feeling confused	6 (6%)
Having a hard time waking up	13 (14%)
Lost consciousness	0 (0%)
Sore throat	59 (62%)
Runny nose	52 (54%)
Nasal congestion	63 (66%)
Muscle pain	45 (47%)
Loss of appetite	32 (33%)
Difficulty sleeping	20 (21%)
Vomiting	10 (10%)
Other	44 (46%)
Did you (or do you) have lingering symptoms (lasting more than 2 weeks)?	52 (55%)
Which lingering symptoms did you experience:	
Chills	0 (0%)
Headache	5 (5 %)
Cough	15 (16%)
Difficulty breathing	4 (4%)
Shortness of breath at rest	5 (5%)
Chest pain	2 (2%)
Feeling confused	2 (2%)
Having a hard time waking up	1 (1%)
Sore throat	0 (0%)
Nasal congestion	7 (7%)
Muscle pain	1 (1%)
Loss of appetite	4 (4%)
Difficulty breathing	4 (4%)
Brain fog	9 (9%)
Memory issues	5 (5%)
Tiredness/fatigue	31 (32%)
Other	23 (24%)
Were you hospitalized because of your infection? (yes)	5 (5%)
Did you receive oxygen therapy by mask or nasal prongs? (yes)	3 (3%)
Did you require intubation or mechanical ventilation? (yes)	0 (0%)

© 2024 Vrantsidis DM et al. *JAMA Network Open.*

eTable 1. Number of Children Reaching Clinical Cutoffs on Each ASQ-3 and ASQ:SE-2 Measure at Ages 12 and 24 Months

	Prenatal SARS-CoV-2 Exposure	No Exposure
Outcome	N (%)	N (%)
Developmental Milestones		
ASQ-3 communication (12 months)		
Above clinical cut-off (not at risk)	73 (91%)	631 (88%)
1 SD below cut-off (requires monitoring)	6 (8%)	67 (9%)
2 SDs below cut-off (clinical referral)	1 (1%)	18 (3%)
ASQ-3 gross motor (12 months)		
Above clinical cut-off (not at risk)	59 (75%)	542 (76%)
1 SD below cut-off (requires monitoring)	12 (15%)	97 (14%)
2 SDs below cut-off (clinical referral)	8 (10%)	77 (11%)
ASQ-3 fine motor (12 months)		
Above clinical cut-off (not at risk)	72 (90%)	655 (92%)
1 SD below cut-off (requires monitoring)	6 (8%)	43 (6%)
2 SDs below cut-off (clinical referral)	2 (3%)	15 (2%)
ASQ-3 problem solving (12 months)		
Above clinical cut-off (not at risk)	66 (84%)	596 (84%)
1 SD below cut-off (requires monitoring)	10 (13%)	73 (10%)
2 SDs below cut-off (clinical referral)	3 (4%)	42 (6%)
ASQ-3 personal-social (12 months)		
Above clinical cut-off (not at risk)	64 (80%)	590 (83%)
1 SD below cut-off (requires monitoring)	6 (8%)	84 (12%)
2 SDs below cut-off (clinical referral)	10 (13%)	38 (5%)
ASQ-3 communication (24 months)		
Above clinical cut-off (not at risk)	54 (83%)	573 (84%)
1 SD below cut-off (requires monitoring)	7 (11%)	53 (8%)
2 SDs below cut-off (clinical referral)	4 (6%)	59 (9%)
ASQ-3 gross motor (24 months)		
Above clinical cut-off (not at risk)	49 (74%)	559 (81%)
1 SD below cut-off (requires monitoring)	14 (22%)	80 (12%)
2 SDs below cut-off (clinical referral)	2 (3%)	47 (7%)
ASQ-3 fine motor (24 months)		
Above clinical cut-off (not at risk)	54 (83%)	597 (87%)
1 SD below cut-off (requires monitoring)	10 (17%)	59 (9%)
2 SDs below cut-off (clinical referral)	0 (0%)	29 (4%)
ASQ-3 problem solving (24 months)		
Above clinical cut-off (not at risk)	50 (77%)	570 (83%)
1 SD below cut-off (requires monitoring)	15 (23%)	92 (13%)
2 SDs below cut-off (clinical referral)	0 (0%)	21 (3%)
ASQ-3 personal-social (24 months)		
Above clinical cut-off (not at risk)	50 (77%)	564 (82%)
1 SD below cut-off (requires monitoring)	12 (19%)	94 (14%)
2 SDs below cut-off (clinical referral)	3 (5%)	27 (4%)

© 2024 Vrantsidis DM et al. *JAMA Network Open.*

Socioemotional Milestones		
ASQ:SE-2 social-emotional (12 months)		
Above clinical cut-off (not at risk)	65 (82%)	543 (77%)
1 SD below cut-off (requires monitoring)	8 (10%)	112 (16%)
2 SDs below cut-off (clinical referral)	6 (8%)	52 (7%)
ASQ:SE-2 social-emotional (24 months)		
Above clinical cut-off (not at risk)	58 (89%)	604 (89%)
1 SD below cut-off (requires monitoring)	6 (9%)	51 (8%)
2 SDs below cut-off (clinical referral)	1 (2%)	20 (3%)

© 2024 Vrantsidis DM et al. *JAMA Network Open.*

eTable 2. Full Model Results, Including Covariates, for Neurodevelopmental Outcomes in Children Exposed Prenatally to SARS-CoV-2 Infection and Those Not Exposed Prenatally

Predictor	b	*p*	95% CI	η_p^2
Temperament				
IBQ-R-VSF surgency (6 months)				
Exposure status	0.09	0.43	-0.13, 0.30	0.00
Pre-pregnancy medical conditions	0.07	0.28	-0.06, 0.21	0.00
SES	-0.01	0.90	-0.11, 0.10	0.00
Overall model fit	F(3, 699) = 0.65	0.58		0.00
IBQ-R-VSF regulation (6 months)				
Exposure status	0.19	**0.03**	0.02, 0.36	0.01
Pre-pregnancy medical conditions	0.10	0.06	-0.00, 0.21	0.01
SES	-0.01	0.74	-0.01, 0.07	0.00
Overall model fit	F(3, 699) = 3.13	**0.03**		0.01
IBQ-R-VSF negative affectivity (6 months)				
Exposure status	-0.01	0.93	-0.27, 0.25	0.00
Pre-pregnancy medical conditions	-0.02	0.80	-0.19, 0.14	0.00
SES	-0.06	0.41	-0.19, 0.08	0.00
Overall model fit	F(3, 692) = 0.23	0.88		0.00
ECBQ negative affectivity (24 months)				
Exposure status	-0.05	0.56	-0.21, 0.11	0.00
Pre-pregnancy medical conditions	0.01	0.84	-0.09, 0.11	0.00
SES	-0.13	**<0.01**	-0.21, -0.05	0.01
Overall model fit	F(3, 754) = 3.90	**0.01**		0.02
Developmental Milestones				
ASQ-3 communication (12 months)				
Exposure status	0.87	0.54	-1.88, 3.61	0.00
Pre-pregnancy medical conditions	-0.42	0.66	-2.28, 1.44	0.00
SES	1.29	0.08	-0.13, 2.71	0.00
Overall model fit	F(3, 795) = 1.36	0.26		0.01
ASQ-3 gross motor (12 months)				
Exposure status	0.59	0.76	-3.14, 4.33	0.00
Pre-pregnancy medical conditions	-1.30	0.31	-3.82, 1.21	0.00
SES	-0.42	0.67	-2.34, 1.50	0.00
Overall model fit	F(3, 794) = 0.39	0.76		0.00
ASQ-3 fine motor (12 months)				

© 2024 Vrantsidis DM et al. *JAMA Network Open.*

Exposure status	-0.79	0.40	-2.63, 1.06	0.00
Pre-pregnancy medical conditions	-0.61	0.34	-1.86, 0.64	0.00
SES	0.47	0.34	-0.49, 1.42	0.00
Overall model fit	F(3, 792) = 1.02	0.38		0.00
ASQ-3 problem solving (12 months)				
Exposure status	-0.44	0.73	-2.97, 2.09	0.00
Pre-pregnancy medical conditions	-0.83	0.34	-2.54, 0.88	0.00
SES	0.59	0.37	-0.71, 1.89	0.00
Overall model fit	F(3, 789) = 0.75	0.52		0.00
ASQ-3 personal-social (12 months)				
Exposure status	-1.21	0.41	-4.07, 1.65	0.00
Pre-pregnancy medical conditions	0.62	0.53	-1.32, 2.56	0.00
SES	0.71	0.34	-0.77, 2.19	0.00
Overall model fit	F(3, 791) = 0.61	0.61		0.00
ASQ-3 communication (24 months)				
Exposure status	0.24	0.89	-3.012, 3.50	0.00
Pre-pregnancy medical conditions	-1.16	0.26	-3.18, 0.87	0.00
SES	3.18	**<0.01**	1.57, 4.80	0.02
Overall model fit	F(3, 749) = 6.11	**<0.01**		0.02
ASQ-3 gross motor (24 months)				
Exposure status	0.61	0.61	-1.73, 2.95	0.00
Pre-pregnancy medical conditions	-1.00	0.18	-2.45, 0.45	0.00
SES	-0.20	0.73	-1.36, 0.96	0.00
Overall model fit	F(3, 750) = 0.68	0.57		0.00
ASQ-3 fine motor (24 months)				
Exposure status	-0.93	0.38	-2.98, 1.13	0.00
Pre-pregnancy medical conditions	-0.97	0.14	-2.25, 0.30	0.00
SES	0.97	0.06	-0.05, 1.99	0.01
Overall model fit	F(3, 749) = 2.68	**0.05**		0.01
ASQ-3 problem solving (24 months)				
Exposure status	-1.68	0.18	-4.14, 0.77	0.00
Pre-pregnancy medical conditions	0.70	0.37	-0.83, 2.22	0.00
SES	1.13	0.07	-0.09, 2.35	0.00
Overall model fit	F(3, 747) = 1.87	0.13		0.01
ASQ-3 personal-social (24 months)				
Exposure status	-1.08	0.35	-3.36, 1.19	0.00
Pre-pregnancy medical conditions	-1.02	0.16	-2.43, 0.39	0.00
SES	1.73	**<0.01**	0.60, 2.85	0.01

© 2024 Vrantsidis DM et al. *JAMA Network Open.*

Overall model fit	F(3, 749) = 4.73	**<0.01**		0.02
Socioemotional Milestones				
ASQ:SE-2 social-emotional (12 months)				
Exposure status	-2.49	0.26	-6.84, 1.86	0.00
Pre-pregnancy medical conditions	-0.85	0.57	-3.79, 2.09	0.00
SES	-3.95	**<0.01**	-6.19, -1.71	0.02
Overall model fit	F(3, 785) = 4.35	**<0.01**		0.02
ASQ:SE-2 social-emotional (24 months)				
Exposure status	-0.58	0.82	-5.55, 4.38	0.00
Pre-pregnancy medical conditions	-0.08	0.96	-3.18, 3.02	0.00
SES	-6.89	**<0.01**	-9.36, -4.42	0.04
Overall model fit	F(3, 739) = 10.26	**<0.01**		0.04

Note: This table provides the full model results for the findings reported in Table 2.

© 2024 Vrantsidis DM et al. *JAMA Network Open.*

eTable 3. Results, Including Covariates, for Developmental Change in Children's Neurodevelopmental Outcomes

Outcome	b (SE)	t	*p*	95% CI
Temperament: Negative affectivity				
Intercept	3.62 (0.04)	96.01	**<0.01**	3.55, 3.70
Exposure status	-0.03 (0.11)	-0.24	0.81	-0.24, 0.19
Timepoint	-0.06 (0.00)	-26.88	**<0.01**	-0.06, -0.05
Exposure × timepoint	-0.00 (0.01)	-0.38	0.70	-0.02, 0.01
Pre-pregnancy medical conditions	-0.00 (0.05)	-0.07	0.94	-0.11, 0.10
SES	-0.09 (0.04)	-2.22	**0.03**	-0.18, -0.01
Developmental Milestones				
Communication				
Intercept	47.26 (0.54)	87.02	**<0.01**	46.20, 48.33
Exposure status	0.99 (1.44)	0.69	0.49	-1.83, 3.81
Timepoint	0.21 (0.04)	4.71	**<0.01**	0.12, 0.29
Exposure × timepoint	-0.05 (0.15)	-0.34	0.73	-0.34, 0.24
Pre-pregnancy medical conditions	-0.63 (0.80)	-0.79	0.43	-2.20, 0.94
SES	2.25 (0.63)	3.60	**<0.01**	1.02, 3.48
Gross motor				
Intercept	45.78 (0.58)	78.85	**<0.01**	44.64, 46.92
Exposure status	0.60 (1.55)	0.39	0.70	-2.43, 3.63
Timepoint	0.62 (0.05)	13.18	**<0.01**	0.53, 0.71
Exposure × timepoint	-0.05 (0.16)	-0.29	0.77	-0.36, 0.26
Pre-pregnancy medical conditions	-1.24 (0.86)	-1.45	0.15	-2.92, 0.44
SES	-0.34 (0.67)	-0.51	0.61	-1.65, 0.97
Fine motor				
Intercept	52.71 (0.35)	150.57	**<0.01**	52.03, 53.40
Exposure status	-0.74 (0.94)	-0.79	0.43	-2.59, 1.10
Timepoint	-0.13 (0.03)	-4.24	**<0.01**	-0.19, -0.07
Exposure × timepoint	0.01 (0.10)	0.05	0.96	-0.20, 0.21
Pre-pregnancy medical conditions	-0.85 (0.50)	-1.70	0.09	-1.83, 0.13
SES	0.71 (0.39)	1.82	0.07	-0.06, 1.48
Problem solving				
Intercept	47.70 (0.45)	105.10	**<0.01**	46.81, 48.59
Exposure status	-0.40 (1.21)	-0.33	0.74	-2.78, 1.97
Timepoint	-0.08 (0.04)	-2.16	**0.03**	-0.16, -0.01
Exposure × timepoint	-0.11 (0.13)	-0.85	0.40	-0.36, 0.14
Pre-pregnancy medical conditions	0.00 (0.66)	0.00	1.00	-1.30, 1.30

© 2024 Vrantsidis DM et al. *JAMA Network Open.*

SES	0.83 (0.52)	1.60	0.11	-0.19, 1.84
Personal-social				
Intercept	44.03 (0.48)	92.53	**<0.01**	43.09, 44.96
Exposure status	-1.14 (1.27)	-0.90	0.37	-3.62, 1.35
Timepoint	0.47 (0.04)	11.48	**<0.01**	0.39, 0.55
Exposure × timepoint	-0.04 (0.14)	-0.30	0.77	-0.31, 0.23
Pre-pregnancy medical conditions	-0.19 (0.69)	-0.27	0.79	-1.54, 1.17
SES	1.17 (0.54)	2.17	**0.03**	0.11, 2.22
Socioemotional Milestones: Social-emotional				
Intercept	29.14 (0.86)	33.98	**<0.01**	27.45, 30.82
Exposure status	-2.65 (2.22)	-1.20	0.23	-7.00, 1.70
Timepoint	-0.15 (0.06)	-2.65	**0.01**	-0.26, -0.04
Exposure × timepoint	0.17 (0.19)	0.85	0.39	-0.22, 0.55
Pre-pregnancy medical conditions	-0.49 (1.32)	-0.37	0.71	-3.08, 2.09
SES	-5.27 (1.03)	-5.14	**<0.01**	-7.28, -3.26

Notes. This table provides the full model results for the findings reported in Table 3.

© 2024 Vrantsidis DM et al. *JAMA Network Open.*

eTable 4. Results Examining Interactions Between Child Sex and Exposure Status

a) Results examining interactions between child sex and exposure status for children's neurodevelopmental outcomes

Predictor	b	*P*	95% CI	η_p^2
Temperament				
IBQ-R-VSF surgency (6 months)				
Exposure status	0.05	0.74	-0.25, 0.34	0.00
Pre-pregnancy medical conditions	0.07	0.30	-0.06, 0.20	0.00
SES	-0.01	0.93	-0.11, 0.10	0.00
Child sex	0.07	0.30	-0.06, 0.19	0.00
Exposure × sex	0.08	0.71	-0.35, 0.51	0.00
Overall model fit	F(5, 699) = 0.71	0.62		0.01
IBQ-R-VSF regulation (6 months)				
Exposure status	0.01	0.94	-0.34, 0.37	0.00
Pre-pregnancy medical conditions	-0.02	0.82	-0.18, 0.15	0.00
SES	-0.06	0.40	-0.19, 0.08	0.00
Child sex	-0.06	0.41	-0.22, 0.09	0.00
Exposure × sex	-0.06	0.83	-0.58, 0.46	0.00
Overall model fit	F(5, 692) = 0.32	0.90		0.00
IBQ-R-VSF negative affectivity (6 months)				
Exposure status	0.14	0.26	-0.10, 0.37	0.00
Pre-pregnancy medical conditions	0.10	0.05	-0.00, 0.21	0.01
SES	-0.01	0.77	-0.10, 0.07	0.00
Child sex	-0.01	0.80	-0.11, 0.09	0.00
Exposure × sex	0.12	0.49	-0.22, 0.46	0.00
Overall model fit	F(5, 699) = 1.97	0.08		0.01
ECBQ negative affectivity (24 months)				
Exposure status	-0.04	0.70	-0.25, 0.17	0.00
Pre-pregnancy medical conditions	0.01	0.82	-0.09, 0.11	0.00
SES	-0.14	**<0.01**	-0.21, -0.06	0.01
Child sex	-0.07	0.13	-0.17, 0.02	0.00
Exposure × sex	-0.02	0.90	-0.34, 0.30	0.00
Overall model fit	F(5, 754) = 2.88	**0.01**		0.02
Developmental Milestones				
ASQ-3 communication (12 months)				
Exposure status	-0.34	0.86	-4.17, 3.50	0.00

© 2024 Vrantsidis DM et al. *JAMA Network Open.*

Pre-pregnancy medical conditions	-0.29	0.76	-2.15, 1.57	0.00
SES	1.25	0.09	-0.17, 2.67	0.00
Child sex	-2.51	**<0.01**	-4.24, -0.77	0.01
Exposure × sex	2.43	0.39	-3.07, 7.92	0.00
Overall model fit	F(5, 795) = 2.42	**0.03**		0.02
ASQ-3 gross motor (12 months)				
Exposure status	-1.51	0.57	-6.71, 3.69	0.00
Pre-pregnancy medical conditions	-1.19	0.35	-3.71, 1.33	0.00
SES	-0.34	0.73	-2.27, 1.58	0.00
Child sex	-0.59	0.62	-2.95, 1.76	0.00
Exposure × sex	4.35	0.26	-3.15, 11.85	0.00
Overall model fit	F(5, 794) = 0.50	0.78		0.00
ASQ-3 fine motor (12 months)				
Exposure status	-1.06	0.42	-3.64, 1.52	0.00
Pre-pregnancy medical conditions	-0.60	0.35	-1.86, 0.65	0.00
SES	0.48	0.32	-0.47, 1.44	0.00
Child sex	0.09	0.88	-1.09, 1.26	0.00
Exposure × sex	0.56	0.77	-3.14, 4.26	0.00
Overall model fit	F(5, 792) = 0.64	0.67		0.00
ASQ-3 problem solving (12 months)				
Exposure status	-0.50	0.78	-4.01, 3.01	0.00
Pre-pregnancy medical conditions	-0.77	0.38	-2.47, 0.94	0.00
SES	0.52	0.44	-0.78, 1.82	0.00
Child sex	-2.12	**0.01**	-3.72, -0.53	0.01
Exposure × sex	0.07	0.98	-4.99, 5.13	0.00
Overall model fit	F(5, 789) = 1.95	0.08		0.01
ASQ-3 personal-social (12 months)				
Exposure status	-3.84	0.06	-7.79, 0.10	0.00
Pre-pregnancy medical conditions	0.87	0.37	-1.05, 2.79	0.00
SES	0.67	0.37	-0.79, 2.13	0.00
Child sex	-4.51	**<0.01**	-6.31, -2.72	0.03
Exposure × sex	5.34	0.06	-0.32, 11.00	0.00
Overall model fit	F(5, 791) = 5.27	**<0.01**		0.03
ASQ-3 communication (24 months)				
Exposure status	-1.04	0.74	-7.09, 5.02	0.00
Pre-pregnancy medical conditions	-0.95	0.53	-3.90, 2.01	0.00
SES	-3.96	**<0.01**	-6.21, -1.71	0.02

© 2024 Vrantsidis DM et al. *JAMA Network Open.*

Child sex	1.42	0.31	-1.34, 4.18	0.00
Exposure × sex	-2.99	0.50	-11.73, 5.75	0.00
Overall model fit	F(5, 749) = 6.82	**<0.01**		0.04
ASQ-3 gross motor (24 months)				
Exposure status	0.37	0.87	-3.98, 4.72	0.00
Pre-pregnancy medical conditions	-1.08	0.29	-3.09, 0.92	0.00
SES	3.13	**<0.01**	1.53, 4.73	0.02
Child sex	-3.57	**<0.01**	-5.46, -1.67	0.02
Exposure × sex	-0.63	0.85	-7.12, 5.86	0.00
Overall model fit	F(5, 750) = 0.55	0.74		0.00
ASQ-3 fine motor (24 months)				
Exposure status	-0.27	0.87	-3.42, 2.88	0.00
Pre-pregnancy medical conditions	-0.97	0.19	-2.42, 0.48	0.00
SES	-0.17	0.78	-1.33, 0.99	0.00
Child sex	-0.02	0.98	-1.39, 1.36	0.00
Exposure × sex	1.97	0.41	-2.73, 6.67	0.00
Overall model fit	F(5, 749) = 1.66	0.14		0.01
ASQ-3 problem solving (24 months)				
Exposure status	-1.24	0.38	-4.01, 1.54	0.00
Pre-pregnancy medical conditions	-0.97	0.14	-2.25, 0.31	0.00
SES	0.99	0.06	-0.04, 2.01	0.00
Child sex	0.18	0.77	-1.03, 1.39	0.00
Exposure × sex	0.72	0.73	-3.42, 4.86	0.00
Overall model fit	F(5, 747) = 3.20	**0.01**		0.02
ASQ-3 personal-social (24 months)				
Exposure status	-3.13	0.06	-6.42, 0.16	0.00
Pre-pregnancy medical conditions	0.80	0.30	-0.72, 2.31	0.00
SES	1.14	0.06	-0.07, 2.36	0.00
Child sex	-2.34	**<0.01**	-3.78, -0.90	0.01
Exposure × sex	3.04	0.22	-1.87, 7.95	0.00
Overall model fit	F(5, 749) = 10.71	**<0.01**		0.07
Socioemotional Milestones				
ASQ:SE-2 social-emotional (12 months)				
Exposure status	-1.03	0.50	-4.02, 1.96	0.00
Pre-pregnancy medical conditions	-0.93	0.19	-2.31, 0.45	0.00
SES	1.66	**<0.01**	0.55, 2.76	0.01
Child sex	-3.90	**<0.01**	-5.21, -2.60	0.04
Exposure × sex	-0.48	0.83	-4.94, 3.98	0.00

© 2024 Vrantsidis DM et al. *JAMA Network Open.*

Overall model fit	F(5, 785) = 2.84	**0.02**		0.02
ASQ:SE-2 social-emotional (24 months)				
Exposure status	-3.61	0.29	-10.30, 3.08	0.00
Pre-pregnancy medical conditions	0.03	0.98	-3.07, 3.13	0.00
SES	-6.78	**<0.01**	-9.26, -4.31	0.04
Child sex	-0.87	0.56	-3.80, 2.07	0.00
Exposure × sex	6.71	0.19	-3.28, 16.69	0.00
Overall model fit	F(5, 739) = 6.51	**<0.01**		0.04

Notes. Child sex was dummy coded as 1 = males and 2 = females with child female sex serving as the reference category in all analyses.

b) Results examining interactions between child sex and exposure status for change in children's neurodevelopmental outcomes

Outcome	b (SE)	T	*p*	95% CI
Temperament: Negative affectivity				
Intercept	48.72 (0.65)	75.21	**<0.01**	47.45, 49.99
Exposure status	0.62 (1.85)	0.33	0.74	-3.01, 4.24
Timepoint	0.21 (0.04)	4.68	**<0.01**	0.12, 0.29
Child sex	-2.99 (0.75)	-3.99	**<0.01**	-4.46, -1.52
Exposure × timepoint	-0.06 (0.15)	-0.38	0.71	-0.35, 0.23
Exposure × sex	0.74 (2.40)	0.31	0.76	-3.98, 5.45
Pre-pregnancy medical conditions	-0.54 (0.80)	-0.68	0.50	-2.10, 1.02
SES	2.17 (0.62)	3.50	**<0.01**	0.95, 3.39
Developmental Milestones				
Communication				
Intercept	45.82 (0.70)	65.65	**<0.01**	44.45, 47.19
Exposure status	-0.55 (1.99)	-0.28	0.78	-4.46, 3.35
Timepoint	0.62 (0.05)	13.17	**<0.01**	0.53, 0.71
Child sex	-0.12 (0.81)	-0.15	0.88	-1.71, 1.47
Exposure × timepoint	-0.04 (0.16)	-0.26	0.79	-0.35, 0.27
Exposure × sex	2.38 (2.60)	0.91	0.36	-2.73, 7.48
Pre-pregnancy medical conditions	-1.20 (0.86)	-1.40	0.16	-2.88, 0.48
SES	-0.29 (0.67)	-0.44	0.66	-1.61, 1.02
Gross motor				
Intercept	52.61 (0.42)	126.09	**<0.01**	51.79, 53.42
Exposure status	-1.06 (1.20)	-0.89	0.37	-3.41, 1.29
Timepoint	-0.13 (0.03)	-4.24	**<0.01**	-0.19, -0.07
Child sex	0.21 (0.47)	0.44	0.66	-0.72, 1.13
Exposure × timepoint	0.01 (0.10)	0.07	0.94	-0.20, 0.21

Exposure × sex	0.66 (1.52)	0.43	0.67	-2.33, 3.64
Pre-pregnancy medical conditions	-0.84 (0.50)	-1.68	0.09	-1.83, 0.14
SES	0.73 (0.39)	1.86	0.06	-0.04, 1.50
Fine motor				
Intercept	48.74 (0.54)	90.09	**<0.01**	47.68, 49.80
Exposure status	-0.98 (1.54)	-0.64	0.52	-4.01, 2.04
Timepoint	-0.08 (0.04)	-2.19	**0.03**	-0.16, -0.01
Child sex	-2.14 (0.62)	-3.46	**<0.01**	-3.36, -0.93
Exposure × timepoint	-0.11 (0.13)	-0.86	0.39	-0.36, 0.14
Exposure × sex	1.15 (2.00)	0.58	0.56	-2.77, 5.07
Pre-pregnancy medical conditions	0.07 (0.66)	0.11	0.91	-1.22, 1.36
SES	0.79 (0.51)	1.53	0.13	-0.22, 1.80
Problem solving				
Intercept	46.04 (0.56)	82.67	**<0.01**	44.95, 47.13
Exposure status	-2.40 (1.60)	-1.51	0.13	-5.53, 0.73
Timepoint	0.47 (0.04)	11.40	**<0.01**	0.39, 0.55
Child sex	-4.15 (0.63)	-6.56	**<0.01**	-5.39, -2.91
Exposure × timepoint	-0.04 (0.14)	-0.29	0.77	-0.31, 0.23
Exposure × sex	2.56 (2.04)	1.26	0.21	-1.44, 6.56
Pre-pregnancy medical conditions	-0.04 (0.67)	-0.06	0.95	-1.36, 1.28
SES	1.10 (0.53)	2.09	**0.04**	0.07, 2.13
Personal-social				
Intercept	29.09 (1.05)	27.78	**<0.01**	27.04, 31.15
Exposure status	-3.61 (2.92)	-1.23	0.22	-9.34, 2.13
Timepoint	-0.15 (0.06)	-2.65	**0.01**	-0.26, -0.04
Child sex	0.05 (1.25)	0.04	0.97	-2.40, 2.50
Exposure × timepoint	0.17 (0.19)	0.87	0.39	-0.21, 0.55
Exposure × sex	1.99 (3.95)	0.50	0.62	-5.76, 9.73
Pre-pregnancy medical conditions	-0.47 (1.32)	-0.35	0.72	-3.06, 2.12
SES	-5.23 (1.03)	-5.08	**<0.01**	-7.25, -3.21
Socioemotional Milestones: Social-emotional				
Intercept	3.66 (0.04)	81.92	**<0.01**	3.57, 3.75
Exposure status	-0.01 (0.13)	-0.08	0.94	-0.27, 0.25
Timepoint	-0.09 (0.00)	-26.90	**<0.01**	-0.09, -0.08
Child sex	-0.07 (0.05)	-1.47	0.14	-0.17, 0.02
Exposure × timepoint	-0.00 (0.01)	-0.40	0.69	-0.03, 0.02
Exposure × sex	-0.03 (0.17)	-0.21	0.83	-0.36, 0.29
Pre-pregnancy medical conditions	-0.00 (0.05)	-0.04	0.97	-0.11, 0.10
SES	-0.10 (0.04)	-2.27	**0.02**	-0.18, -0.01

© 2024 Vrantsidis DM et al. *JAMA Network Open.*

Notes. Child sex was dummy coded as 1 = males and 2 = females with child female sex serving as the reference category in all analyses.

© 2024 Vrantsidis DM et al. *JAMA Network Open.*

eTable 5. Analyses by Trimester and Severity of Exposure

a) Descriptive statistics for neurodevelopmental outcomes for children exposed prenatally to SARS-CoV-2 by trimester of exposure

Outcome	Trimester 1		Trimester 2		Trimester 3	
	N	M (SD)	N	M (SD)	N	M (SD)
Temperament						
IBQ-R-VSF surgency (6 months)	18	4.76 (0.88)	25	4.81 (0.75)	17	4.69 (0.72)
IBQ-R-VSF regulation (6 months)	18	5.56 (0.68)	25	5.72 (0.40)	17	5.53 (0.50)
IBQ-R-VSF negative affectivity (6 months)	18	3.84 (1.02)	25	3.22 (0.98)	17	3.84 (1.14)
ECBQ negative affectivity (24 months)	14	2.72 (0.61)	31	2.50 (0.51)	23	2.52 (0.68)
Developmental Milestones						
ASQ-3 communication (12 months)	19	48.68 (8.79)	36	49.67 (8.78)	25	46.60 (12.31)
ASQ-3 gross motor (12 months)	19	43.16 (16.18)	35	45.86 (16.06)	25	48.20 (15.80)
ASQ-3 fine motor (12 months)	19	52.11 (8.22)	36	50.83 (8.58)	25	53.20 (5.93)
ASQ-3 problem solving (12 months)	19	46.58 (11.43)	35	47.29 (10.10)	25	48.20 (10.79)
ASQ-3 personal-social (12 months)	19	43.42 (10.28)	36	43.19 (14.65)	25	42.60 (14.44)
ASQ-3 communication (24 months)	14	56.07 (7.64)	30	48.50 (12.47)	21	47.86 (11.13)
ASQ-3 gross motor (24 months)	14	55.00 (8.09)	30	53.83 (6.39)	21	50.95 (8.16)
ASQ-3 fine motor (24 months)	14	51.79 (7.50)	30	49.67 (6.69)	21	49.05 (7.35)
ASQ-3 problem solving (24 months)	14	48.21 (9.53)	30	43.50 (9.30)	21	45.48 (7.05)
ASQ-3 personal-social (24 months)	14	53.57 (5.35)	30	47.50 (10.73)	21	46.43 (8.68)
Socioemotional Milestones						
ASQ:SE-2 social-emotional (12 months)	19	26.05 (17.53)	35	24.14 (18.17)	25	26.00 (15.94)
ASQ:SE-2 social-emotional (24 months)	14	25.00 (13.45)	30	24.50 (15.11)	21	27.86 (17.93)

© 2024 Vrantsidis DM et al. *JAMA Network Open.*

b) ANCOVAs for neurodevelopmental outcomes for children exposed prenatally to SARS-CoV-2 by trimester of exposure

Predictor	b	*p*	95% CI	η_p^2
Temperament				
IBQ-R-VSF surgency (6 months)				
Trimester of exposure (1st vs. 2nd)	0.15	0.54	-0.34, 0.64	0.01
Trimester of exposure (1st vs. 3rd)	0.01	0.98	-0.53, 0.54	0.00
Pre-pregnancy medical risk	0.38	0.08	-0.04, 0.81	0.06
SES	0.19	0.24	-0.13, 0.51	0.03
Overall model fit	F(4, 55) = 1.05	0.39		0.07
IBQ-R-VSF regulation (6 months)				
Trimester of exposure (1st vs. 2nd)	0.20	0.23	-0.13, 0.53	0.03
Trimester of exposure (1st vs. 3rd)	-0.03	0.88	-0.39, 0.34	0.00
Pre-pregnancy medical risk	0.24	0.10	-0.05, 0.53	0.05
SES	-0.01	0.96	-0.22, 0.21	0.00
Overall model fit	F(4, 55) = 1.15	0.34		0.08
IBQ-R-VSF negative affectivity (6 months)				
Trimester of exposure (1st vs. 2nd)	-0.54	0.11	-1.19, 0.12	0.05
Trimester of exposure (1st vs. 3rd)	0.12	0.74	-0.60, 0.84	0.00
Pre-pregnancy medical risk	0.10	0.72	-0.47, 0.68	0.00
SES	0.35	0.11	-0.08, 0.78	0.05
Overall model fit	F(4, 55) = 1.96	0.11		0.13
ECBQ negative affectivity (24 months)				
Trimester of exposure (1st vs. 2nd)	-0.23	0.25	-0.62, 0.17	0.02
Trimester of exposure (1st vs. 3rd)	-0.25	0.23	-0.67, 0.16	0.02
Pre-pregnancy medical risk	0.15	0.32	-0.15, 0.45	0.02
SES	-0.19	0.12	-0.44, 0.05	0.04
Overall model fit	F(4, 63) = 1.36	0.26		0.08
Developmental Milestones				
ASQ-3 communication (12 months)				
Trimester of exposure (1st vs. 2nd)	1.25	0.67	-4.60, 7.09	0.00
Trimester of exposure (1st vs. 3rd)	-1.54	0.63	-7.86, 4.78	0.00
Pre-pregnancy medical risk	0.43	0.86	-4.53, 5.39	0.00
SES	1.53	0.44	-2.38, 5.44	0.01
Overall model fit	F(4, 75) = 0.49	0.74		0.03
ASQ-3 gross motor (12 months)				
Trimester of exposure (1st vs. 2nd)	0.63	0.89	-8.50, 9.75	0.00
Trimester of exposure (1st vs. 3rd)	2.67	0.59	-7.16, 12.50	0.00
Pre-pregnancy medical risk	-7.71	0.05	-15.44, 0.02	0.05
SES	-3.81	0.22	-9.90, 2.27	0.02
Overall model fit	F(4, 74) = 1.49	0.22		0.07
ASQ-3 fine motor (12 months)				
Trimester of exposure (1st vs. 2nd)	-0.95	0.67	-5.46, 3.55	0.00
Trimester of exposure (1st vs. 3rd)	1.74	0.48	-3.13, 6.61	0.01
Pre-pregnancy medical risk	0.54	0.78	-3.28, 4.36	0.00
SES	1.80	0.24	-1.21, 4.81	0.02

© 2024 Vrantsidis DM et al. *JAMA Network Open.*

Overall model fit	F(4, 75) = 0.70	0.59		0.04
ASQ-3 problem solving (12 months)				
Trimester of exposure (1st vs. 2nd)	0.86	0.78	-5.29, 7.01	0.00
Trimester of exposure (1st vs. 3rd)	2.48	0.46	-4.15, 9.10	0.01
Pre-pregnancy medical risk	-0.85	0.75	-6.06, 4.36	0.00
SES	3.16	0.13	-0.94, 7.27	0.03
Overall model fit	F(4, 74) = 0.74	0.57		0.04
ASQ-3 personal-social (12 months)				
Trimester of exposure (1st vs. 2nd)	-0.38	0.93	-8.39, 7.63	0.00
Trimester of exposure (1st vs. 3rd)	-1.15	0.79	-9.81, 7.51	0.00
Pre-pregnancy medical risk	-0.21	0.95	-7.01, 6.58	0.00
SES	-0.95	0.72	-6.31, 4.41	0.00
Overall model fit	F(4, 75) = 0.04	1.00		0.00
ASQ-3 communication (24 months)				
Trimester of exposure (1st vs. 2nd)	-7.14	0.06	-14.73, 0.45	0.06
Trimester of exposure (1st vs. 3rd)	-7.42	0.07	-15.45, 0.61	0.05
Pre-pregnancy medical risk	-0.67	0.82	-6.56, 5.21	0.00
SES	2.46	0.31	-2.38, 7.30	0.02
Overall model fit	F(4, 60) = 1.63	0.18		0.10
ASQ-3 gross motor (24 months)				
Trimester of exposure (1st vs. 2nd)	-1.63	0.52	-6.66, 3.40	0.01
Trimester of exposure (1st vs. 3rd)	-4.40	0.10	-9.72, 0.92	0.04
Pre-pregnancy medical risk	-1.29	0.51	-5.19, 2.61	0.01
SES	-0.47	0.77	-3.68, 2.74	0.00
Overall model fit	F(4, 60) = 0.85	0.50		0.05
ASQ-3 fine motor (24 months)				
Trimester of exposure (1st vs. 2nd)	-2.12	0.39	-6.97, 2.73	0.01
Trimester of exposure (1st vs. 3rd)	-2.77	0.28	-7.91, 2.36	0.02
Pre-pregnancy medical risk	0.13	0.94	-3.63, 3.90	0.00
SES	-0.15	0.93	-3.24, 2.95	0.00
Overall model fit	F(4, 60) = 0.33	0.86		0.02
ASQ-3 problem solving (24 months)				
Trimester of exposure (1st vs. 2nd)	-4.79	0.11	-10.75, 1.163	0.04
Trimester of exposure (1st vs. 3rd)	-2.75	0.39	-9.05, 3.55	0.01
Pre-pregnancy medical risk	-0.42	0.86	-5.04, 4.20	0.00
SES	0.14	0.94	-3.66, 3.93	0.00
Overall model fit	F(4, 60) = 0.70	0.59		0.04
ASQ-3 personal-social (24 months)				
Trimester of exposure (1st vs. 2nd)	-6.20	0.04	-12.26, -0.15	0.07
Trimester of exposure (1st vs. 3rd)	-6.54	0.05	-12.95, -0.13	0.06
Pre-pregnancy medical risk	-3.16	0.18	-7.86, 1.53	0.03
SES	2.92	0.14	-0.94, 6.78	0.04
Overall model fit	F(4, 60) = 2.67	**0.04**		0.15
Socioemotional Milestones				
ASQ:SE-2 social-emotional (12 months)				
Trimester of exposure (1st vs. 2nd)	-1.93	0.70	-11.80, 7.93	0.00

© 2024 Vrantsidis DM et al. *JAMA Network Open.*

Trimester of exposure (1st vs. 3rd)	-1.62	0.76	-12.25, 9.01	0.00
Pre-pregnancy medical risk	3.10	0.46	-5.26, 11.46	0.01
SES	-6.56	0.05	-13.14, 0.02	0.05
Overall model fit	F(4, 74) = 1.34	0.26		0.07
ASQ:SE-2 social-emotional (24 months)				
Trimester of exposure (1st vs. 2nd)	-0.88	0.87	-11.48, 9.72	0.00
Trimester of exposure (1st vs. 3rd)	1.66	0.77	-9.56, 12.87	0.00
Pre-pregnancy medical risk	2.59	0.53	-5.63, 10.82	0.01
SES	-4.35	0.20	-11.11, 2.41	0.03
Overall model fit	F(4, 60) = 0.73	0.58		0.05

c) Descriptive statistics for neurodevelopmental outcomes for children exposed prenatally to SARS-CoV-2 by SARS-CoV-2 infection severity

Outcome	N	M (SD)
Temperament		
IBQ-R-VSF surgency (6 months)	60	2.58 (0.81)
IBQ-R-VSF regulation (6 months)	60	2.58 (0.81)
IBQ-R-VSF negative affectivity (6 months)	60	2.58 (0.81)
ECBQ negative affectivity (24 months)	68	2.43 (0.72)
Developmental Milestones		
ASQ-3 communication (12 months)	80	2.46 (0.69)
ASQ-3 gross motor (12 months)	79	2.46 (0.69)
ASQ-3 fine motor (12 months)	80	2.46 (0.69)
ASQ-3 problem solving (12 months)	79	2.46 (0.69)
ASQ-3 personal-social (12 months)	80	2.46 (0.69)
ASQ-3 communication (24 months)	65	2.45 (0.73)
ASQ-3 gross motor (24 months)	65	2.45 (0.73)
ASQ-3 fine motor (24 months)	65	2.45 (0.73)
ASQ-3 problem solving (24 months)	65	2.45 (0.73)
ASQ-3 personal-social (24 months)	65	2.45 (0.73)
Socioemotional Milestones		
ASQ:SE-2 social-emotional (12 months)	79	2.46 (0.69)
ASQ:SE-2 social-emotional (24 months)	65	2.450.73)

d) ANCOVAs for neurodevelopmental outcomes for children exposed prenatally to SARS-CoV-2 by SARS-CoV-2 infection severity

Predictor	b	*p*	η_p^2	95% CI
Temperament				
IBQ-R-VSF surgency (6 months)				
Infection severity	0.18	0.31	0.02	-0.17, 0.52
Pre-pregnancy medical risk	0.29	0.23	0.03	-0.19, 0.78
SES	0.23	0.11	0.04	-0.05, 0.51
Overall model fit	F(3, 50) = 1.91	0.14	0.09	
IBQ-R-VSF regulation (6 months)				

Infection severity	-0.05	0.71	0.00	-0.34, 0.23
Pre-pregnancy medical risk	0.23	0.18	0.03	-0.11, 0.56
SES	-0.02	0.81	0.00	-0.22, 0.17
Overall model fit	F(3, 50) = 0.85	0.47	0.04	
IBQ-R-VSF negative affectivity (6 months)				
Infection severity	0.12	0.52	0.01	-0.26, 0.51
Pre-pregnancy medical risk	0.15	0.64	0.00	-0.49, 0.78
SES	0.39	0.21	0.03	-0.23, 1.00
Overall model fit	F(3, 50) = 1.10	0.36	0.06	
ECBQ negative affectivity (24 months)				
Infection severity	0.11	0.24	0.02	-0.08, 0.30
Pre-pregnancy medical risk	0.15	0.35	0.01	-0.17, 0.48
SES	-0.14	0.33	0.02	-0.43, 0.14
Overall model fit	F(3, 50) = 1.65	0.19	0.07	
Developmental Milestones				
ASQ-3 communication (12 months)				
Infection severity	-0.41	0.87	0.00	-5.24, 4.43
Pre-pregnancy medical risk	0.35	0.90	0.00	-5.09, 5.80
SES	1.71	0.30	0.01	-1.54, 4.96
Overall model fit	F(3, 50) = 0.31	0.82	0.01	
ASQ-3 gross motor (12 months)				
Infection severity	-3.96	0.16	0.03	-9.54, 1.62
Pre-pregnancy medical risk	-7.35	0.07	0.04	-15.38, 0.69
SES	-5.04	**0.04**	0.05	-9.84, -0.23
Overall model fit	F(3, 50) = 2.72	0.05	0.10	
ASQ-3 fine motor (12 months)				
Infection severity	0.52	0.68	0.00	-1.99, 3.02
Pre-pregnancy medical risk	0.56	0.76	0.00	-3.02, 4.13
SES	1.62	0.35	0.01	-1.84, 5.08
Overall model fit	F(3, 50) = 0.41	0.75	0.02	
ASQ-3 problem solving (12 months)				
Infection severity	0.28	0.88	0.00	-3.28, 3.85
Pre-pregnancy medical risk	-1.11	0.70	0.00	-6.90, 4.69
SES	2.88	0.14	0.03	-0.95, 6.71
Overall model fit	F(3, 50) = 0.80	0.50	0.03	
ASQ-3 personal-social (12 months)				
Infection severity	-0.23	0.93	0.00	-5.06, 4.61
Pre-pregnancy medical risk	-0.09	0.98	0.00	-7.17, 6.99
SES	-0.84	0.74	0.00	-5.95, 4.27
Overall model fit	F(3, 50) = 0.03	0.99	0.00	
ASQ-3 communication (24 months)				

© 2024 Vrantsidis DM et al. *JAMA Network Open.*

Infection severity	1.71	0.46	0.01	-2.87, 6.29
Pre-pregnancy medical risk	-0.01	1.00	0.00	-6.37, 6.34
SES	3.85	**0.04**	0.07	0.12, 7.58
Overall model fit	F(3, 50) = 0.98	0.41	0.05	
ASQ-3 gross motor (24 months)				
Infection severity	-0.64	0.69	0.00	-3.87, 2.59
Pre-pregnancy medical risk	-0.87	0.68	0.00	-5.11, 3.37
SES	0.01	1.00	0.00	-3.76, 3.77
Overall model fit	F(3, 50) = 0.18	0.91	0.01	
ASQ-3 fine motor (24 months)				
Infection severity	0.02	0.99	0.00	-2.58, 2.63
Pre-pregnancy medical risk	0.47	0.81	0.00	-3.39, 4.33
SES	0.26	0.89	0.00	-3.51, 4.03
Overall model fit	F(3, 50) = 0.03	0.99	0.00	
ASQ-3 problem solving (24 months)				
Infection severity	0.81	0.67	0.00	-3.00, 4.62
Pre-pregnancy medical risk	0.19	0.94	0.00	-4.70, 5.09
SES	0.76	0.71	0.00	-3.33, 4.85
Overall model fit	F(3, 50) = 0.14	0.94	0.01	
ASQ-3 personal-social (24 months)				
Infection severity	1.49	0.31	0.02	-1.40, 4.38
Pre-pregnancy medical risk	-2.60	0.32	0.02	-7.76, 2.57
SES	4.14	**0.01**	0.10	0.92, 7.37
Overall model fit	F(3, 50) = 2.06	0.11	0.09	
Socioemotional Milestones				
ASQ:SE-2 social-emotional (12 months)				
Infection severity	2.54	0.32	0.01	-2.47, 7.55
Pre-pregnancy medical risk	3.07	0.52	0.01	-6.30, 12.45
SES	-5.84	0.06	0.05	-11.92, 0.24
Overall model fit	F(3, 50) = 2.04	0.12	0.08	
ASQ:SE-2 social-emotional (24 months)				
Infection severity	-1.54	0.50	0.01	-6.07, 2.99
Pre-pregnancy medical risk	3.31	0.41	0.01	-4.71, 11.34
SES	-4.74	0.08	0.05	-10.02, 0.54
Overall model fit	F(3, 50) = 0.98	0.41	0.05	

© 2024 Vrantsidis DM et al. *JAMA Network Open.*

eTable 6. Results Based on Imputed Data

a) Pooled descriptive statistics based on 50 imputed data sets

	Prenatal SARS-CoV-2 Exposure (n=96)		No Exposure (n=800)	
Outcome	N missing data (%)	M (SD)	N missing data (%)	M (SD)
Temperament				
IBQ-R-VSF surgency (6 months)	36 (38%)	4.72	160 (20%)	4.68
IBQ-R-VSF regulation (6 months)	36 (38%)	5.59	160 (20%)	5.42
IBQ-R-VSF negative affectivity (6 months)	36 (38%)	3.58	167 (21%)	3.61
ECBQ negative affectivity (24 months)	28 (29%)	2.53	113 (14%)	2.58
Developmental Milestones				
ASQ-3 communication (12 months)	16 (17%)	48.33	84 (11%)	47.60
ASQ-3 gross motor (12 months)	17 (18%)	45.87	84 (11%)	45.30
ASQ-3 fine motor (12 months)	16 (17%)	51.66	87 (11%)	52.61
ASQ-3 problem solving (12 months)	17 (18%)	47.36	89 (11%)	47.81
ASQ-3 personal-social (12 months)	16 (17%)	43.00	88 (11%)	44.19
ASQ-3 communication (24 months)	31 (32%)	50.12	115 (14%)	50.29
ASQ-3 gross motor (24 months)	17 (18%)	52.89	114 (14%)	52.72
ASQ-3 fine motor (24 months)	31 (32%)	50.15	115 (14%)	51.13
ASQ-3 problem solving (24 months)	31 (32%)	44.96	117 (15%)	46.96
ASQ-3 personal-social (24 months)	31 (32%)	48.52	115 (14%)	49.92
Socioemotional Milestones				
ASQ:SE-2 social-emotional (12 months)	31 (32%)	25.37	93 (12%)	25.75
ASQ:SE-2 social-emotional (24 months)	31 (32%)	25.62	125 (16%)	27.68

Note. No participants were missing infection status or covariate data.

b) Pooled ANCOVA results based on 50 imputed data sets

Predictor	b	*p*	95% CI
Temperament			
IBQ-R-VSF surgency (6 months)			
Exposure status	0.04	0.70	-0.17, 0.25
Pre-pregnancy medical conditions	0.09	0.18	-0.04, 0.22
SES	-0.02	0.75	-0.12, 0.08
IBQ-R-VSF regulation (6 months)			
Exposure status	0.17	**0.05**	0.00, 0.33
Pre-pregnancy medical conditions	0.11	**0.04**	0.01, 0.22
SES	-0.03	0.52	-0.11, 0.06

© 2024 Vrantsidis DM et al. *JAMA Network Open.*

IBQ-R-VSF negative affectivity (6 months)			
Exposure status	-0.03	0.81	-0.30, 0.23
Pre-pregnancy medical conditions	0.02	0.86	-0.15, 0.18
SES	-0.03	0.67	-0.17, 0.11
ECBQ negative affectivity (24 months)			
Exposure status	-0.07	0.39	-0.23, 0.09
Pre-pregnancy medical conditions	0.01	0.85	-0.09, 0.11
SES	-0.12	**<0.01**	-0.20, -0.04
Developmental Milestones			
ASQ-3 communication (12 months)			
Exposure status	0.86	0.53	-1.82, 3.54
Pre-pregnancy medical conditions	-0.23	0.80	-2.09, 1.62
SES	1.22	0.10	-0.22, 2.67
ASQ-3 gross motor (12 months)			
Exposure status	0.55	0.77	-3.17, 4.28
Pre-pregnancy medical conditions	-1.49	0.25	-4.03, 1.05
SES	-0.55	0.57	-2.45, 1.34
ASQ-3 fine motor (12 months)			
Exposure status	-0.89	0.35	-2.76, 0.97
Pre-pregnancy medical conditions	-0.54	0.40	-1.81, 0.73
SES	0.49	0.32	-0.48, 1.46
ASQ-3 problem solving (12 months)			
Exposure status	-0.38	0.77	-2.97, 2.21
Pre-pregnancy medical conditions	-0.64	0.47	-2.37, 1.08
SES	0.54	0.42	-0.78, 1.86
ASQ-3 personal-social (12 months)			
Exposure status	-1.15	0.43	-4.02, 1.72
Pre-pregnancy medical conditions	0.71	0.47	-1.24, 2.67
SES	0.56	0.46	-0.91, 2.03
ASQ-3 communication (24 months)			
Exposure status	0.18	0.91	-3.02, 3.37
Pre-pregnancy medical conditions	-1.17	0.25	-3.15, 0.81
SES	3.11	**<0.01**	1.51, 4.70
ASQ-3 gross motor (24 months)			
Exposure status	0.17	0.89	-2.18, 2.52
Pre-pregnancy medical conditions	-1.09	0.14	-2.51, 0.34
SES	-0.27	0.64	-1.40, 0.86
ASQ-3 fine motor (24 months)			
Exposure status	-0.88	0.40	-2.93, 1.17
Pre-pregnancy medical conditions	-1.00	0.12	-2.25, 0.25
SES	0.78	0.12	-0.21, 1.76
ASQ-3 problem solving (24 months)			
Exposure status	-1.92	0.12	-4.311, 0.48
Pre-pregnancy medical conditions	0.72	0.35	-0.79, 2.23
SES	1.04	0.09	-0.16, 2.24
ASQ-3 personal-social (24 months)			

© 2024 Vrantsidis DM et al. *JAMA Network Open.*

Exposure status	-1.21	0.31	-3.57, 1.14
Pre-pregnancy medical conditions	-1.10	0.12	-2.46, 0.27
SES	1.54	**0.01**	0.42, 2.65
Socioemotional Milestones			
ASQ:SE-2 social-emotional (12 months)			
Exposure status	-2.68	0.22	-6.96, 1.59
Pre-pregnancy medical conditions	-0.84	0.57	-3.74, 2.05
SES	-3.93	**<0.01**	-6.15, -1.71
ASQ:SE-2 social-emotional (24 months)			
Exposure status	-0.78	0.75	-5.56, 4.00
Pre-pregnancy medical conditions	-0.01	1.00	-3.09, 3.07
SES	-6.55	**<0.01**	-9.02, -4.08

Notes. F-statistics are not computed for imputed data sets in SPSS.

c) Pooled mixed model results based on 50 imputed data sets

Outcome	b (SE)	t	*p*	95% CI
Temperament: Negative affectivity				
Intercept	3.62 (0.04)	94.50	**<0.01**	3.55, 3.70
Exposure status	-0.02 (0.10)	-0.15	0.88	-0.22, 0.19
Timepoint	-0.06 (0.00)	-24.10	**<0.01**	-0.06, -0.05
Exposure × timepoint	0.00 (0.01)	-0.28	0.78	-0.02, 0.01
Pre-pregnancy medical conditions	-0.01 (0.05)	-0.12	0.90	-0.11, 0.10
SES	-0.09 (0.04)	-2.28	**0.02**	-0.17, -0.01
Developmental Milestones				
Communication				
Intercept	47.42 (0.55)	86.93	**<0.01**	46.35, 48.49
Exposure status	0.89 (1.41)	0.63	0.53	-1.87, 3.64
Timepoint	0.20 (0.05)	4.21	**<0.01**	0.11, 0.29
Exposure × timepoint	-0.07 (0.15)	-0.44	0.66	-0.36, 0.23
Pre-pregnancy medical conditions	-0.74 (0.79)	-0.92	0.36	-2.29, 0.82
SES	2.19 (0.61)	3.58	**<0.01**	0.99, 3.38
Gross motor				
Intercept	45.79 (0.57)	79.79	**<0.01**	44.66, 46.91
Exposure status	0.57 (1.53)	0.37	0.71	-2.43, 3.57
Timepoint	0.61 (0.05)	11.81	**<0.01**	0.51, 0.71
Exposure × timepoint	0.00 (0.17)	-0.01	0.99	-0.33, 0.32
Pre-pregnancy medical conditions	-1.15 (0.83)	-1.38	0.17	-2.78, 0.48
SES	-0.37 (0.64)	-0.57	0.57	-1.63, 0.90
Fine motor				
Intercept	52.73 (0.35)	151.86	**<0.01**	52.05, 53.41

Exposure status	-0.75 (0.91)	-0.82	0.41	-2.54, 1.04
Timepoint	-0.14 (0.03)	-4.13	**<0.01**	-0.20, -0.07
Exposure × timepoint	-0.01 (0.10)	-0.12	0.90	-0.22, 0.19
Pre-pregnancy medical conditions	-0.76 (0.48)	-1.57	0.12	-1.70, 0.19
SES	0.71 (0.38)	1.89	0.06	-0.03, 1.45
Problem solving				
Intercept	47.74 (0.45)	105.35	**<0.01**	46.85, 48.62
Exposure status	-0.59 (1.24)	-0.48	0.63	-3.03, 1.84
Timepoint	-0.09 (0.04)	-2.13	**0.03**	-0.17, -0.01
Exposure × timepoint	-0.07 (0.13)	-0.55	0.58	-0.34, 0.19
Pre-pregnancy medical conditions	-0.04 (0.66)	-0.06	0.95	-1.33, 1.25
SES	0.81 (0.51)	1.60	0.11	-0.18, 1.80
Personal-social				
Intercept	44.05 (0.47)	93.27	**<0.01**	43.13, 44.98
Exposure status	-1.14 (1.25)	-0.92	0.36	-3.58, 1.30
Timepoint	0.46 (0.04)	10.78	**<0.01**	0.38, 0.54
Exposure × timepoint	-0.03 (0.13)	-0.20	0.84	-0.29, 0.23
Pre-pregnancy medical conditions	-0.16 (0.67)	-0.23	0.81	-1.48, 1.16
SES	1.14 (0.53)	2.16	**0.03**	0.10, 2.17
Socioemotional Milestones: Social-emotional				
Intercept	28.87 (0.85)	33.92	**<0.01**	27.20, 30.54
Exposure status	-2.61 (2.26)	-1.16	0.25	-7.05, 1.82
Timepoint	-0.15 (0.06)	-2.28	**0.02**	-0.28, -0.02
Exposure × timepoint	0.16 (0.22)	0.74	0.46	-0.27, 0.59
Pre-pregnancy medical conditions	-0.61 (1.28)	-0.47	0.64	-3.12, 1.91
SES	-5.29 (0.98)	-5.38	**<0.01**	-7.22, -3.37

Notes. F-statistics are not computed for imputed data sets in SPSS.

© 2024 Vrantsidis DM et al. *JAMA Network Open.*
