# Supplementary material for: Neurodevelopment in the First 2 Years of Life Following Prenatal Exposure to Maternal SARS-CoV-2 Infection
Source: JAMA Netw Open. 2024 Nov 7;7(11):e2443697. doi: 10.1001/jamanetworkopen.2024.43697 (PMC11544495; doi:10.1001/jamanetworkopen.2024.43697)
Supplement: Supplement 2. — Data Sharing Statement [file jamanetwopen-e2443697-s002.pdf]

Data Sharing Statement

Vrantsidis. Neurodevelopment in the First 2 Years of Life Following Prenatal Exposure to Maternal SARS-CoV-2 Infection. *JAMA Netw Open*. Published November 07, 2024. doi:10.1001/jamanetworkopen.2024.43697

Data
Data available: No

Additional Information
Explanation for why data not available: Data are available upon reasonable request.
